# Evaluation of Cross-Protocol Stability of a Fully Automated Brain Multi-Atlas Parcellation Tool

**DOI:** 10.1371/journal.pone.0133533

**Published:** 2015-07-24

**Authors:** Zifei Liang, Xiaohai He, Can Ceritoglu, Xiaoying Tang, Yue Li, Kwame S. Kutten, Kenichi Oishi, Michael I. Miller, Susumu Mori, Andreia V. Faria

**Affiliations:** 1 College of Electronics and Information Engineering, Sichuan University, Chengdu, China; 2 Department of Biomedical Engineering, Johns Hopkins University School of Medicine, Baltimore, Maryland, United States of America; 3 The Russell H. Morgan Department of Radiology and Radiological Science, Johns Hopkins University School of Medicine, Baltimore, Maryland, United States of America; 4 Center for Imaging Science, Johns Hopkins University, Baltimore, Maryland, United States of America; Chinese Academy of Sciences, CHINA

## Abstract

Brain parcellation tools based on multiple-atlas algorithms have recently emerged as a promising method with which to accurately define brain structures. When dealing with data from various sources, it is crucial that these tools are robust for many different imaging protocols. In this study, we tested the robustness of a multiple-atlas, likelihood fusion algorithm using Alzheimer’s Disease Neuroimaging Initiative (ADNI) data with six different protocols, comprising three manufacturers and two magnetic field strengths. The entire brain was parceled into five different levels of granularity. In each level, which defines a set of brain structures, ranging from eight to 286 regions, we evaluated the variability of brain volumes related to the protocol, age, and diagnosis (healthy or Alzheimer’s disease). Our results indicated that, with proper pre-processing steps, the impact of different protocols is minor compared to biological effects, such as age and pathology. A precise knowledge of the sources of data variation enables sufficient statistical power and ensures the reliability of an anatomical analysis when using this automated brain parcellation tool on datasets from various imaging protocols, such as clinical databases.

## Introduction

It is a widely accepted notion that brain anatomy, delineated by MRI, carries clinically important information to support diagnosis and medical decisions. For patients with suspected neurodegenerative diseases, MRI is an important clinical tool, with which physicians evaluate the anatomy subjectively, using their knowledge accumulated through education and experience, and reach the best possible medical decisions. Probably, the most important role of MRI for these patient populations is to rule out tumor and stroke [1–3], which usually cause large anatomical changes. For this purpose, with the current image analysis technologies, the ability of human judgment is considered more reliable than automated detection tools. Even after tumor and/or stroke are ruled out, MRI data still contain a wealth of anatomical information that could be medically informative. These include changes in volume or shape, based on which the level of atrophy of specific brain structures could be evaluated, and changes in intensity, such as hyper intense ischemic lesions seen in T2-weighted and Fluid Attenuated Inversion Recovery (FLAIR) images. Unlike tumor and stroke, in which abnormal features appear that should not exist in normal brains, the shape and intensity changes caused by many neurodegenerative diseases, especially in the early stage or preclinical stage, are an extension of a continuum from a normal range of anatomical variability and age-dependent changes. With the sheer number of brain structures and image voxels, it could be argued that the human ability to capture the anatomical features and relate these to clinical outcomes is limited. Indeed, our current knowledge about the relationship between anatomical features and clinically important information, such as diagnosis, functional loss, or prognosis, is not strong enough to allow MRI to play more than a small role in medical decision-making and patient care for neurodegenerative diseases and dementia populations [1–3].

A widely used research paradigm in brain MRI is to use quantitative image analysis to quantify anatomical features, perform correlation analysis with clinical information, and eventually find important features (i.e., biomarkers or surrogate markers) that cannot be well appreciated by human perception alone. This paradigm has supported numerous studies in the past, in which an analysis based on image normalization, such as voxel-based analysis, was used in many studies (for review, see[4]). Based on specific anatomical features that are linked to a patient group of interest, successful discrimination of diseases, such as Alzheimer’s disease, has been reported [5], including studies using ADNI data [6–8]. While successful, the application of these approaches in routine clinical practice, however, would face several challenges. This is partly due to fundamental differences in the study design. Specifically, in research settings, the patient population is highly homogenized (so that the abnormality exists in common anatomical locations among the patient group), image protocols are consistent (so that subtle changes can be consistently quantified), a control population of high quality is available (so that the normal range of anatomical variability and bias in the population averages are minimized), and, eventually, group comparisons can be performed with proper statistical power. All these factors are not the case in clinical practice. Indeed, the initial stratification of the highly heterogeneous patient population is one of the most important missions of clinical MRI, and eventually, a judgment must be made for each individual patient. The heart of this clinical paradigm is the knowledge-versus-individual, not the group-versus-group study design, in which knowledge information from all individuals is retained without a reduction into group-based statistics (group-aggregated statistics cannot be used for a heterogeneous population) [9].

If we want to create a quantitative knowledge database of anatomical phenotypes, there are several interesting questions to be addressed. First, it is uncertain whether raw images with more than one million voxels are suitable as the contents of such a database; the sheer amount of the noisy voxel information could severely hamper our ability to store, search, and analyze the anatomical contents and perform a group vs. individual analysis. Second, such a large database inevitably needs to contain data from multiple sources and the tools that extract anatomical information need to be robust for a reasonable range of variability in scanning protocols. As long as the protocol effects cannot be completely eliminated, it is also important to know the range of measurement variability that would enable estimation of the abnormality-detection power, which would include both biological and artifactual (protocol differences, hardware performance, quantification accuracy) contributions.

In this paper, we have extended past efforts to convert the dense imagery information into a set of structural representations by brain parcellation tools [10–14]. We are especially interested in the performance of the multiple-atlas brain parcellation approach against protocol differences. The brain parcellation by the multiple-atlas is a newly emerging technique [15–23]. In this study, we adopted a new multi-atlas fusion algorithm that is based on the advanced diffeomorphic image transformation and utilizes both the location and intensity information of each structure for likelihood estimation [24]. In addition, we employed an atlas library with a novel hierarchical structure definition technique [25]. Although there are many past studies measuring the accuracy of the multi-atlas approaches, there are fewer studies addressing the precision (reproducibility) of the tools. In the multi-atlas analysis, the atlases serve as a spatial filter that teaches the algorithms how voxels should be aggregated to define structures. Obviously, there are numerous ways to define structures (thus aggregate voxels) and, thus, accuracy and precision of the tools depend on the atlases. In addition, as the atlas libraries are inevitably foreign data for the majority of the users with different imaging protocols, estimation of the bias due to protocol differences is of great importance. This was tested using ADNI control data from six different protocols with three different manufacturers and two different field strengths. We also used ADNI AD data for pathology effect analysis. All analyses were performed based on our fully automated T1-image analysis pipeline in the MriCloud platform (www.mricloud.org). Based on this analysis, we measured the impact of protocol differences on the parcellation results, and compared its extent with two types of biological effect sizes: age and AD pathology.

## Methods and Materials

### Subjects

The data used in the preparation of this article, summarized in Table 1, were obtained from the Alzheimer’s Disease Neuroimaging Initiative (ADNI) database (adni.loni.usc.edu). The normal data (Control, n = 72) from ADNI with six different types of hardware (GE, Siemens, and Philips, all with both 1.5T and 3T) were used to evaluate the effect size of the image protocols and subject ages. For patient populations, the AD populations were obtained from ADNI AD data (n = 48).

**Table 1:** Imaging parameters of the six protocols.

Group	Age	Scanner manufacturer	Field Strength(T)	Voxel size	Number of subjects
**normal**	70–90	**Philips**	1.5	1.2x0.94x0.94	12
**normal**	59–95	**Philips**	3	1.2x1x1	12
**normal**	65–96	**SIEMENS**	1.5	1.2x1.25x1.25	12
**normal**	56–91	**SIEMENS**	3	1.2x1.x1	12
**normal**	70–94	**GE**	1.5	1.2x0.94x0.94	12
**normal**	64–88	**GE**	3	1.2x1.02x1.02	12
**AD**	59–85	**Philips**	1.5	1.2x0.94x0.94	7
**AD**	56–85	**Philips**	3	1.2x1x1	8
**AD**	56–93	**SIEMENS**	1.5	1.2x1.25x1.25	7
**AD**	56–93	**SIEMENS**	3	1.2x1.x1	9
**AD**	55–92	**GE**	1.5	1.2x0.94x0.94	9
**AD**	64–93	**GE**	3	1.2x1.02x1.02	8

The ADNI was launched in 2003 by the National Institute on Aging (NIA), the National Institute of Biomedical Imaging and Bioengineering (NIBIB), the Food and Drug Administration (FDA), private pharmaceutical companies, and non-profit organizations, as a $60 million, five-year public/private partnership. The primary goal of ADNI has been to test whether serial magnetic resonance imaging (MRI), positron emission tomography (PET), other biological markers, and clinical and neuropsychological assessment can be combined to measure the progression of mild cognitive impairment (MCI) and early Alzheimer’s disease (AD). Determination of sensitive and specific markers of very early AD progression is intended to aid researchers and clinicians in developing new treatments and monitoring the effectiveness of these treatments, as well as reducing the time and cost of clinical trials. The Principal Investigator of this initiative is Michael W. Weiner, MD, VA Medical Center and University of California–San Francisco. ADNI is the result of the efforts of many co-investigators from a broad range of academic institutions and private corporations, and subjects have been recruited from over 50 sites across the U.S. and Canada. The initial goal of ADNI was to recruit 800 subjects, but ADNI has been followed by ADNI-GO and ADNI-2. To date, these three protocols have recruited over 1500 adults, ages 55 to 90, to participate in the research, consisting of cognitively normal older individuals, people with early or late MCI, and people with early AD. The follow-up duration of each group is specified in the protocols for ADNI-1, ADNI-2, and ADNI-GO. Subjects originally recruited for ADNI-1 and ADNI-GO had the option to be followed in ADNI-2. For up-to-date information, see www.adni-info.org.

### Image Processing

**Fig 1:**
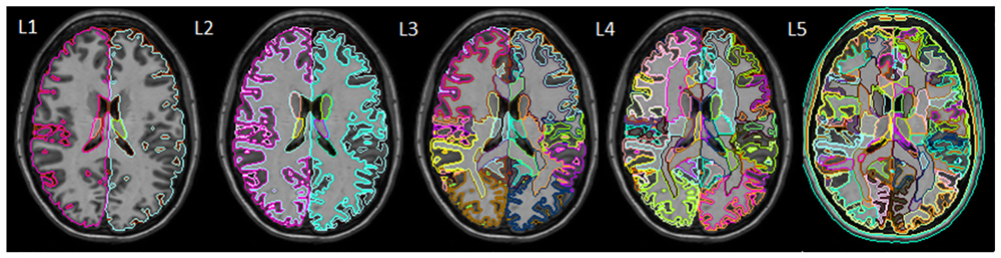
Brain parcellation scheme of the JHU multiple atlases. Multiple granularity levels (L1 to L5) are shown. Level 5 (L5) has the highest granularity and defines 286 regions. An anatomy-based hierarchical relationship was established to generate super-structures and lower-granularity parcellation, as shown in L1–L4.

Three-dimension (3D), magnetization-prepared, rapid gradient-echo (MPRAGE) sagittal images were used to evaluate brain morphology. The images were parcellated by an automated pipeline deployed at https://www.mricloud.org/, a platform that is open to the public after registration. The pipeline uses large diffeomorphic deformation metric mapping (LDDMM) and multi-atlas likelihood fusion (MALF) algorithms [24,26,27]. Twenty-three atlases (JHU adult atlas Version 5J) were used, in which the 286 structures were defined with a five-level ontological hierarchical relationship, as described by [25]. For the multiple-granularity analysis, the 286 defined structures were combined hierarchically to generate five different levels of anatomical representations. The numbers of the structures defined at each level were 8, 19, 53, 125, and 286 for levels 1 to 5, respectively (Fig 1). This pipeline accepts raw images without pre-processing, such as skull-stripping, matrix size, and orientation adjustment, intensity matching, or homogeneity correction. After the parcellation, the volumes of the defined structures were quantified.

The raw volume data (S3 Table) contains a large amount of cross-subject variability. For example, the average of the total brain volume of the entire dataset (n = 120) was 1,223,823 mm^3^ +/- 128,184 mm^3^. The coefficient of the variance of the 286 structures was 20 +/- 15.5%. This was expected because brain size is known to be highly variable. In order to minimize the effect of this variation, we normalized the volume of each structure based on the total brain volume, which was calculated from the volumes of the tissue, the ventricles, and the sulci. After this operation, the coefficient of the variance was reduced to 16+/- 11.5%. This, however, by no means indicates that normalized values should always be used for quantitative analyses.

### Multi-atlas pipeline

Although all data used in this study were acquired by MPRAGE, the employed parameters had considerable differences in terms of the image dimensions (e.g., fields of view, matrix sizes, voxel sizes, etc.) and time-domain parameters (e.g., scanning times, inversion recovery time, echo time, etc.), in addition to the hardware differences (manufacturers, magnetic field strength, the number of channels, etc). The detailed protocol information can be found at http://www.adni-info.org/scientists/ADNIStudyProcedures.aspx. These differences led to variability in image contrasts and signal-to-noise ratios. Especially troublesome was the considerable amount of variability in spatial intensity inhomogeneity. Because MPRAGE is not a quantitative imaging method, the image intensity is an arbitrary unit. Thus, pre-processing of the images, including intensity-matching and homogeneity correction, is essential to minimize the influence of protocol differences. We found that it is extremely difficult to perform accurate intensity-matching and homogeneity correction for images of the entire head, which includes signals from many regions outside the brain that tend to have severe intensity inhomogeneity due to radio-frequency fall-off, in addition to a large amount of cross-subject variability, such as the level of lipids.

Our pipeline employed a three-step pre-processing routine. In the first step, we pre-generated a population-averaged atlas created from the 23 atlases using linear transformation in the MNI space, from which the probabilistic brain location was obtained. The subject image was also linearly registered to the MNI space and brain-masking was applied using the probabilistic map. The histogram-matching and homogeneity correction by the N4 algorithm were then applied [28]. In the second step, the nonlinear transformation between the subject image and the 23 atlases was performed using Large Deformation Diffeomorphic Metric Mapping (LDDMM) [26]. Based on the transformation matrices obtained from the nonlinear transformation, the structural labels were transferred from the 23 atlases to the subjects. In the third step, the potential mismatch of signal intensity between the subject image and the 23 atlases were probed using the intensity reports of the defined structures, and potential bias fields were created and applied to the atlas images. Then, in a second hierarchical level, the second LDDMM was performed and the structural labels were again applied to the subjects. In this study, we used three steps LDDMM, with alphas—that represent the degree of elasticity of the mapping—of 0.05, 0.003, 0.002.

The label of each voxel in the final step was determined by the MALF algorithm; please read [27]for details. The estimation is formulated in the Bayesian setting[24], enabling a natural incorporation of various types of information, such as intensity profile, the structural positions within the brain (preserved under the action of diffeomorphism), and the anatomical topology inherited from the manually-defined atlases. The estimation of the parameter of interest is performed via maximum a posteriori estimation using the expectation-maximization (EM) algorithm. The likelihoods of multiple atlases are fused in the E-step while the optimal estimator, a single maximizer of the fused likelihoods, is then obtained in the M-step. This two-level hierarchical segmentation pipeline is completely automated and has been implemented in the MriCloud platform (www.mricloud.org). The computations were processed on the Gordon cluster of XSEDE[29]. Each node on Gordon contains two 8-core 2.6 GHz Intel EM64T Xeon E5 (Sandy Bridge) processors and 64 GB of DDR3-1333 memory. The total segmentation time of one subject is around 40 min for an atlas set with 23 atlases using 4 nodes/64 cores.

Previously, we validated this segmentation algorithm on datasets with various disease models and anatomical states (age, disease model and status, image quality, magnetic field) [27]. The Dice overlap between our methods and manually defined structures (gold-standard) ranged between 0.8–0.96, indicating a high level of accuracy. In comparisons with other widely used segmentation tools (Freesurfer and FSL) and multi-atlas algorithms (STAPLE, Spatial STAPLE, ANTS+PICSL), our pipeline showed comparative or superior accuracy.

### Statistical Analysis

We tested the normalized volumes of each structure defined by our automated parcellation tool for (i) differences among protocols, (ii) age-dependence, and (iii) differences related to the diagnosis. For (i) and (ii), we included healthy subjects only (“controls” in Table 1, n = 72), in order to avoid any uncontrolled disease effect. The differences among protocols (six groups: [GE, Philips, Siemens] x [1.5T, 3.0T], see Table 1) and diagnosis (AD or control, n = 120) were tested by ANOVA. For the age-dependence analysis, the data from controls were pooled and the age effect was examined by Pearson regression. The p-values were corrected by a Bonferroni threshold set at p<0.05.

## Results

### Protocol effect

**Fig 2:**
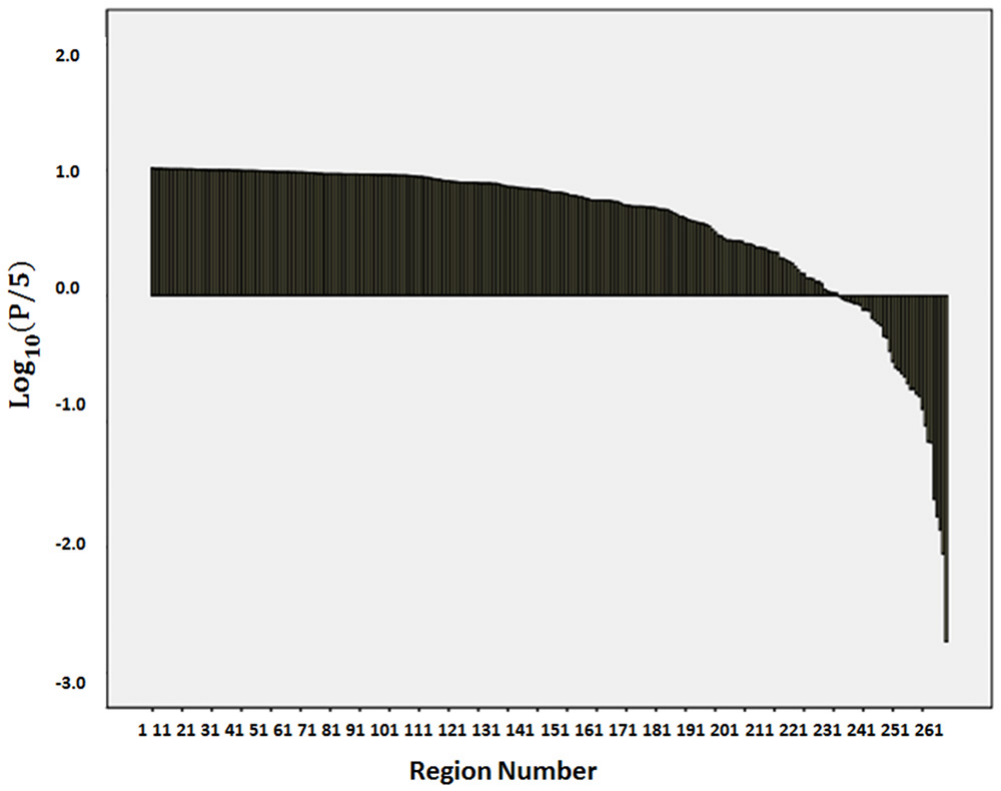
Distribution of Bonferroni-corrected p-values for protocol differences in 286 structures at Level 5. For better visualization, the p-values (P, y-axis) are presented as Log_10_(P/5). A p-value of 0.05 corresponds to -2 on the y-axis. At this threshold, two regions reached statistical significance.

**Fig 3:**
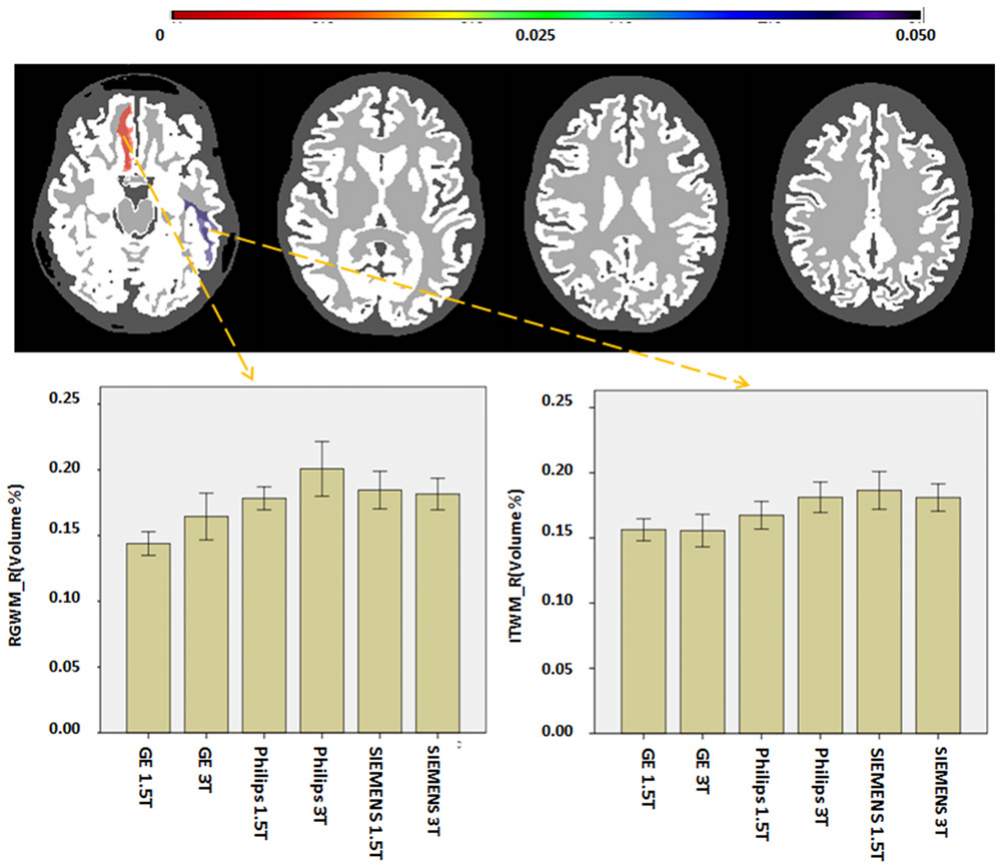
Regional volumes affected by the differences in protocol. The volumes are normalized by the “total brain” volume (parenchyma plus CSF). Two regions (left inferior temporal gyrus [ITWM_L] and the right rectus gyrus [RGWM_R] white matter) showed significantly smaller volumes on the GE scanners.

We examined protocol-dependent bias in the volume measurements at the five different granularity levels. The volumes of the defined structures did not show significant differences in any of the six protocols at the levels of granularity 1 to 4. At these levels, the lowest p-values were 0.5184, 0.3869, 0.6264, and 0.1565, respectively, suggesting an insignificant influence of the protocols. Fig 2 shows the distribution of Bonferroni-corrected p-values of the 286 structures at level 5. At this highest granularity level, two regions reached statistical significance. The actual regions and inter-protocol differences are shown in Fig 3. These regions were the white matter of the left inferior temporal (ITWM_L, p-value = 0.04) and right rectus gyrus (RGWM_R, p-value = 0.008), in which GE scanners tend to have smaller values. These regions constitute approximately 0.35% and 0.15% of the total brain volume, respectively.

### Age effect

**Fig 4:**
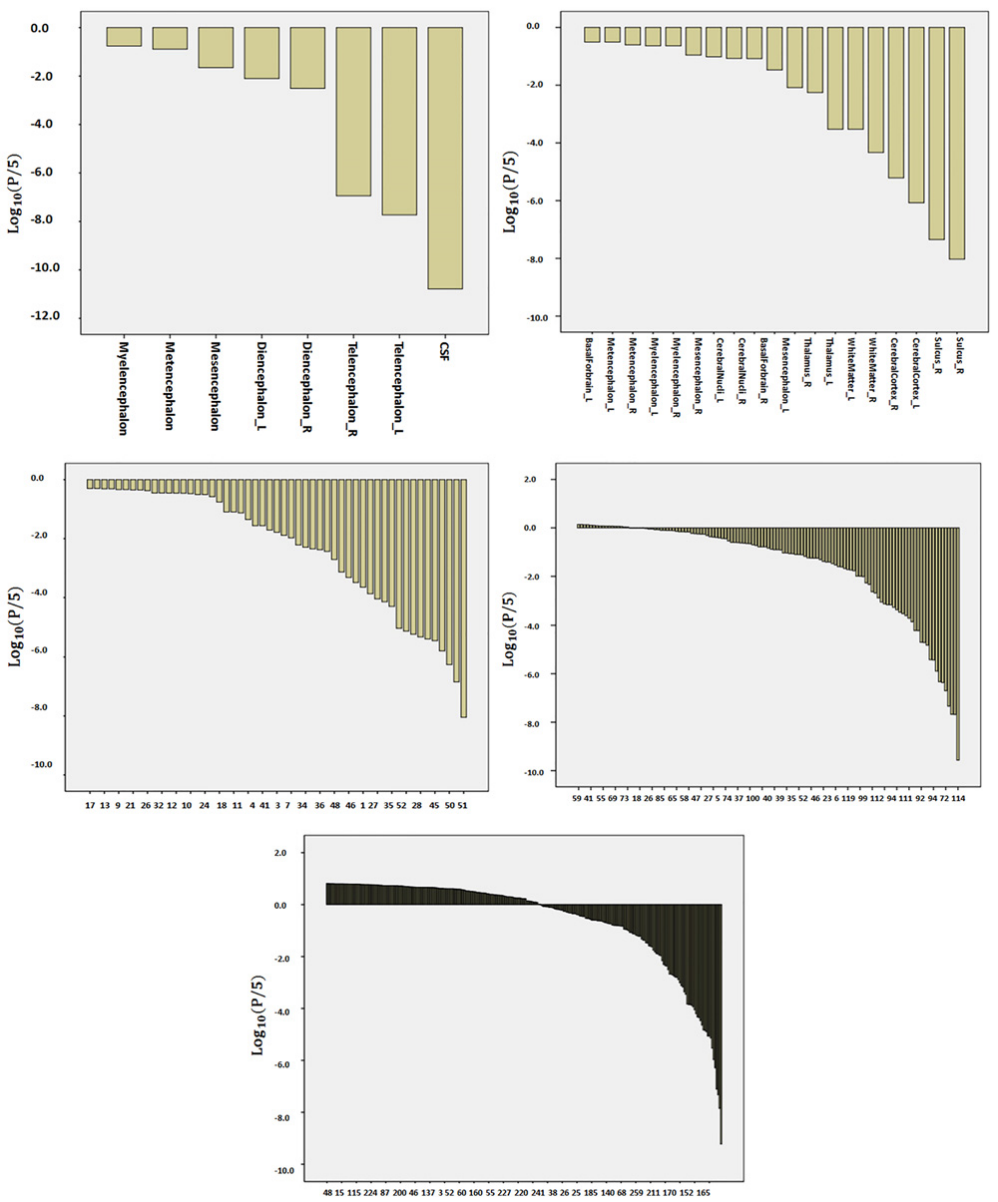
Distribution of Bonferroni-corrected p-values from the correlation between age and regional volumes. All the granularity levels are shown. For better visualization, the P values are presented as Log_10_(P/5). Therefore, p<0.05 corresponds to <-2 on the y-axis.

**Fig 5:**
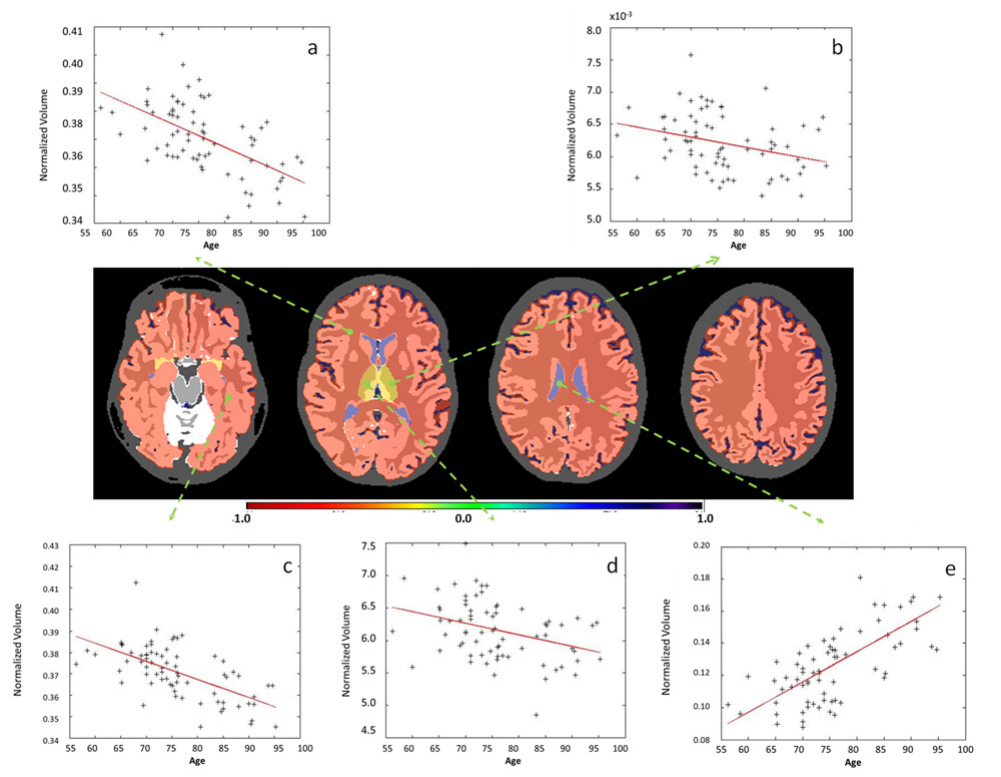
Examples of significant correlations between regional volumes and age at granularity level 1. The colors code the correlation coefficient R in regions where the age vs. volume curve achieves significance (p-value <0.05, Bonferroni-corrected). Yellow / orange / red (R<0) are regions that shrink over time, while blue (R>0) are regions that expand over time, such as ventricles. As is shown in the figure: a. telencephalon (right); b. diencephalon (left); c. telencephalon (left); d. diencephalon (right); e. cerebrospinal fluid.

The influence of a protocol is meaningful only when compared with the biological effect size, which, in the present study, is represented by the age. To examine this biological effect, all the data from controls of the six different protocols were pooled, and the age-dependent changes in structural volumes were evaluated. Fig 4 and S1 Table show the p-values from the regression between the age and volumes of the defined structures at each granularity level. Fig 5 shows examples of significant age-dependent changes in volumes. At level 1, compartments with cerebrospinal fluid (CSF), the telencephalon, and the diencephalon reached statistical significance (Log_10_(P/5) < -2.0, i.e., corrected P<0.05). The largest effects were seen for the reductions of the telencephalon (hemispheres) over age, which accompanied significant enlargement of the CSF areas that include the ventricles and sulci.

**Fig 6:**
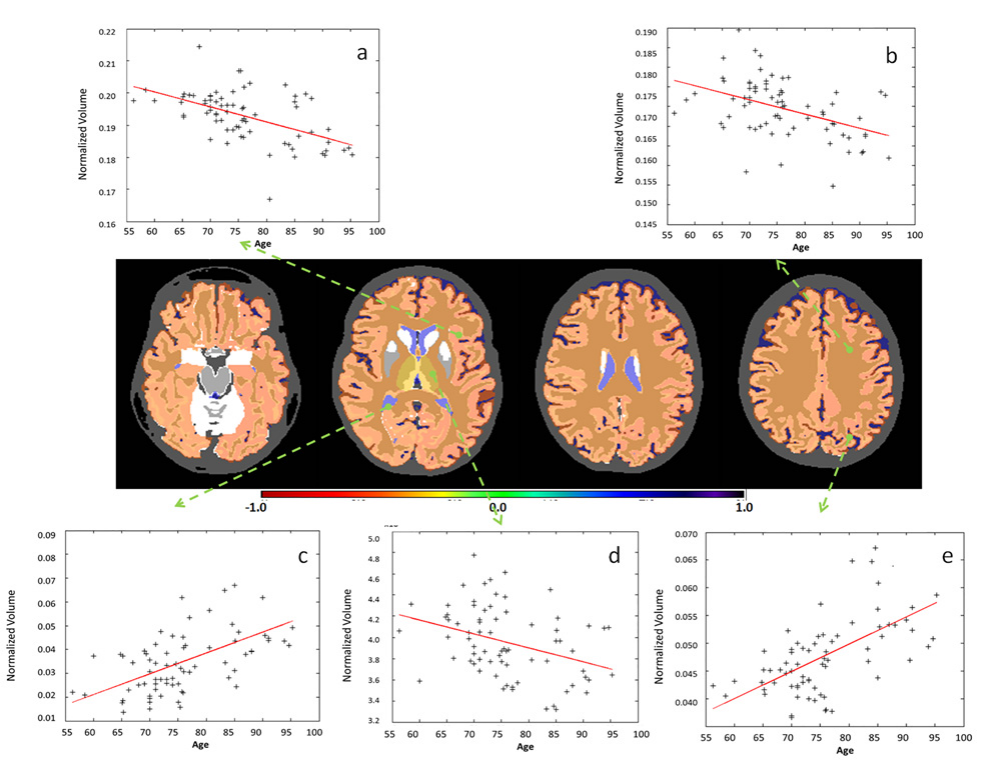
Examples of significant correlations between regional volumes and age at granularity level 2. The colors code the correlation coefficient R in regions where the age vs. volume curve achieves significance (p-value <0.05, Bonferroni-corrected). Yellow / orange / red (R<0) are regions that shrink over time, while blue (R>0) are regions that expand over time, such as ventricles. As is shown in the figure: a. cerebral cortex (left); b. white matter (left); c. ventricle; d. thalamus (left); e. sulcus (left).

**Fig 7:**
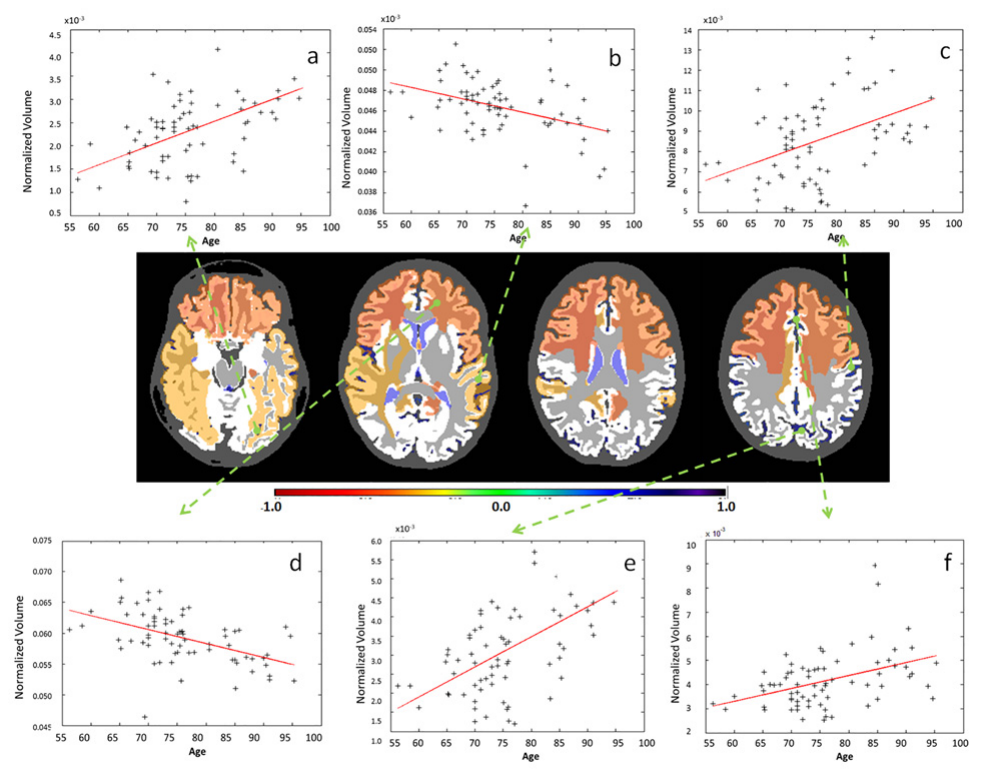
Examples of significant correlations between regional volumes and age at granularity level 3. The colors code the correlation coefficient R in regions where the age vs. volume curve achieves significance (p-value <0.05, Bonferroni-corrected). Yellow / orange / red (R<0) are regions that shrink over time, while blue (R>0) are regions that expand over time, such as ventricles. As is shown in the figure: a. sulci of the temporal lobe (left); b. temporal lobe (left); c. sulci of the parietal lobe (left); d. anterior part of the white matter (left); e.sulci of the occipital lobe (left); f. sulci of the cingulate gyrus (left).

**Fig 8:**
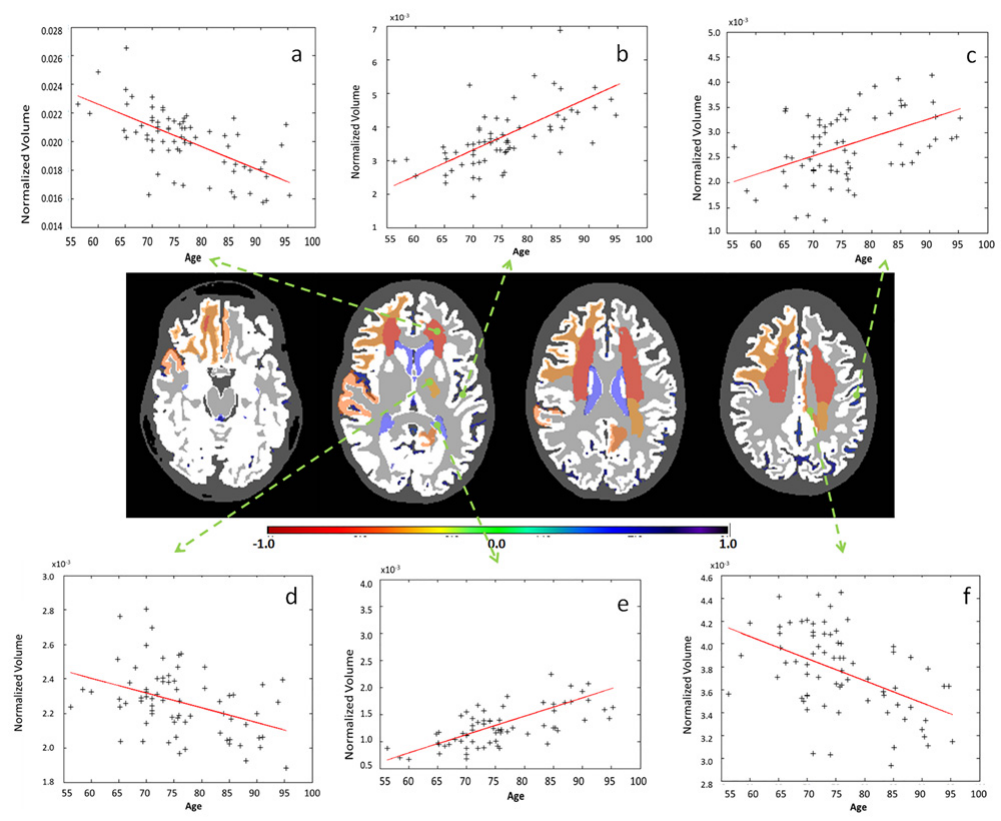
Examples of significant correlations between regional volumes and age at granularity level 4. The colors code the correlation coefficient R in regions where the age vs. volume curve achieves significance (p-value <0.05, Bonferroni-corrected). Yellow / orange / red (R<0) are regions that shrink over time, while blue (R>0) are regions that expand over time, such as ventricles. As is shown in the figure: a. anterior part of the deep and periventricular white matter (left); b. Sylvian fissure and posterior insular sulcus (left); c. central sulcus (left); d. posterior limb of the internal capsule (left); e. Inferior part of the lateral ventricle (left); f. subcortical white matter of the cingulate gyrus (left).

**Fig 9:**
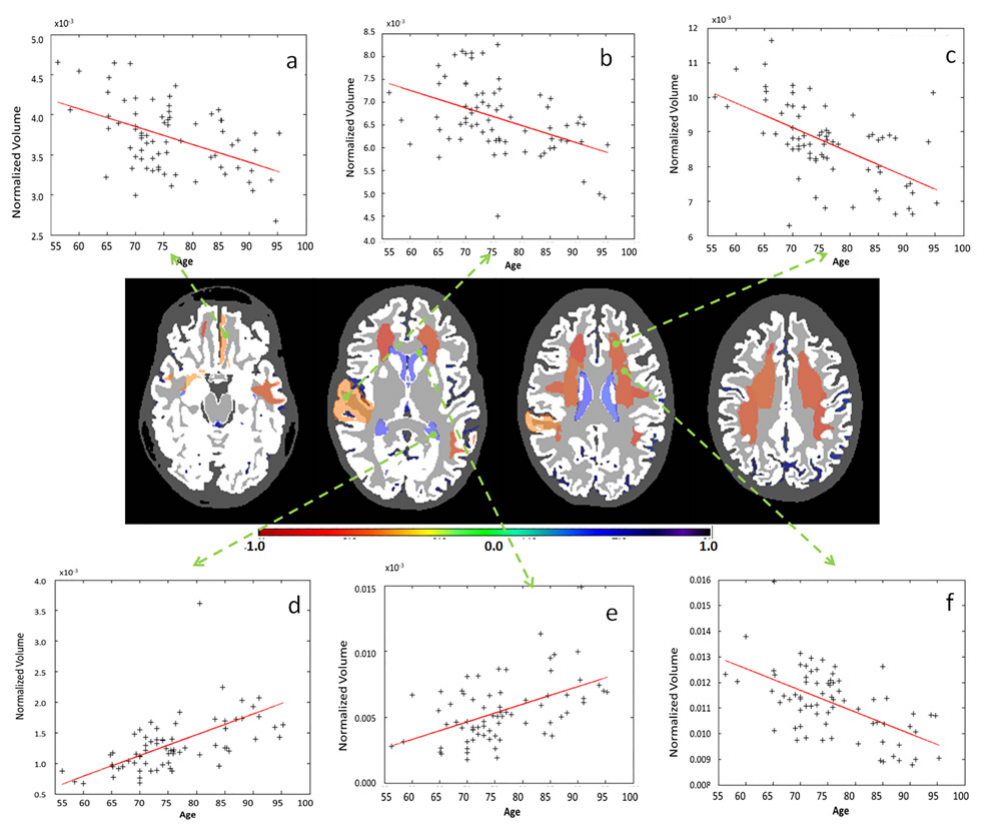
Examples of significant correlations between regional volumes and age at granularity level 5. The colors code the correlation coefficient R in regions where the age vs. volume curve achieves significance (p-value <0.05, Bonferroni-corrected). Yellow / orange / red (R<0) are regions that shrink over time, while blue (R>0) are regions that expand over time, such as ventricles. As is shown in the figure: a. gryus rectus (left); b. subcortical white matter of the superior temporal gyrus (right); c. anterior corona radiata (left); d. inferior horn of the lateral ventricle (left); e. frontal horn of the lateral ventricle (left); superior corona radiata (left).

**Fig 10:**
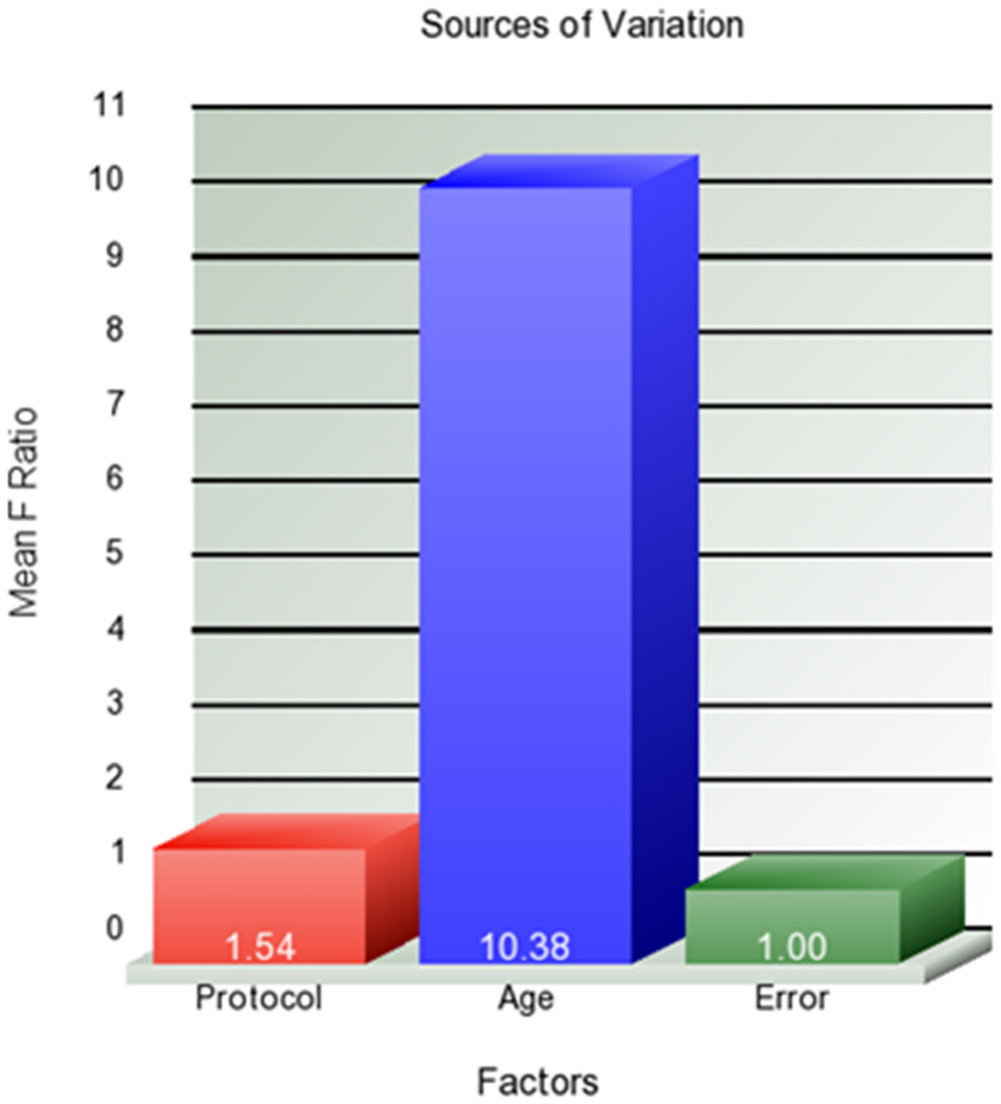
Source of variation in normalized regional volumes at level 5. More than 10% of variance is explained by age, 1.54% is explained by the protocols, and 1% is attributed to error.

As the granularity increased and the volumes of defined structures decreased, the number of areas with a significant correlation with age decreased, revealing spatial specificity of the age effect at the bilateral frontal and temporal lobes (Figs 6, 7, 8 and 9). At level 5, the white matter regions in the frontal lobes, the ventricles, and a small section in the temporal lobes reached a significant level. The analysis of the source of variation for level 5 is shown in Fig 10. Age explained 10.38% of the total variation in the data, which was much larger than the variation attributed to protocols (1.53%) and the error (1%).

### Diagnosis analysis

Similarly to the age-dependence analysis, all the data from different protocols were pooled and divided into the AD groups and age-matched control groups. The regions with significant differences between groups (p-value < 0.05 after the Bonferroni correction) are highlighted in S2 Table. The inspection of regions with a significant amount of atrophy through the different levels of granularity revealed AD-specific anatomical features. In level 1, atrophy of the telencephalon and enlargement of the ventricles and sulci were significant. Moving up the granularity levels, the atrophic areas were further specified to the gray matter rather than the white matter (level 2), and temporal and limbic gray matter structures (level 3 and 4), while focal atrophy of the white matter in the temporal lobes was also identified (levels 4 and 5). At the highest level, structures such as the temporal gyri, the hippocampus, and related gyri, the fornix, and the cingulum reached significance, as expected.

## Discussion

### Use of ADNI data

We examined the robustness of a multi-atlas brain parcellation tool in light of differences in imaging protocols. This type of analysis may not be crucial in classical research designs, in which both patient and control groups are typically scanned under the same protocol. However, if we are interested in creating a large knowledge database, integration of data from various sources with different protocols becomes an important issue. Of course, we cannot expect an automated parcellation tool to be robust against, for example, high-resolution MPRAGE and spin-echo-based T1-weighted images with 10mm slice thicknesses; a reasonable amount of consistency in the imaging protocols is expected. In this respect, we believe that the “ADNI T1 protocol,” with somewhat loosely defined parameters, provides a good representation of the protocol variability in modern clinical practice.

In this study, we used 120 cases from ADNI data with six different protocols. This sample size, which is relevant to typical MRI-based studies, defines the amount of biological effects we can detect. Our study indicated that the effect sizes of age and atrophy of AD patients could be detected with the substantial amount of the protocol differences. However, it should be noted that smaller pathological effects, such as those expected in various psychiatric diseases, could be obscured by the noise and/or biases introduced by the protocol differences and far larger sample sized would be needed for the detection.

### Impact of protocols using the multi-atlas approach

It is likely that the protocol effect will differ depending on the algorithm for the automated parcellation. In this study, we used a multi-atlas approach, in which we used our 23 atlases as teaching files to identify structures. Interestingly, the knowledge-based judgment and cross-protocol issues also apply to the multi-atlas tool, in general. Namely, the multiple atlases with pre-parcellated structural definitions are, in many cases, external data for application studies. In this study, our atlases were developed using our internal data acquired on Philips scanners, which were external to the ADNI data. Although it is theoretically possible to build study-specific multiple atlases, the extensive whole-brain segmentation involved in this process makes the development of specific multiple atlases prohibitively time-consuming.

In our multi-atlas pipeline, we employed a three-step processing approach to ensure accurate image intensity-matching and homogeneity correction. The final labeling step was based on MALF, which uses both the location and intensity information to determine the voxel labeling. In this type of multi-atlas labeling, several label candidates relevant to the location compete for a given voxel, incorporating the intensity information from the 23 atlases. We expect this final labeling step would also contribute to the robustness of the results against protocol differences, as long as the target structure has a distinctive intensity signature.

Another consideration is the number of atlases to be used. It is true that the number of atlases positively correlates with the accuracy of the segmentation, but at the cost of computational power and time. Aljabar, et al. [3] showed that the results start to converge when using more than 10 atlases. In this study, we used the total set of 23 atlases, which was well above this number. However, it is not clear whether the optimum number of the atlases is fixed; factors such the age, gender, and anatomical features of the atlases affect the results. For example, the number of atlases required to achieve the same level of accuracy for a young adult may not be the same for an atlas set that contains only a young adult (e.g. 20–30 yrs) and another set that covers the wider range (e.g. 20–90 yrs).

### Evaluation of protocol differences and age effect

An ideal way to evaluate protocol effects, both related to image acquisition and post-processing, is to scan the same subjects with different protocols. However, the variability of such data must be interpreted within the context of biological effects of interest. This would require scanning many subjects at different biological states; e.g., age distributions and AD pathology, which would make a “same subjects” study prohibitively difficult. In our “different subjects” study, the observed variability was a mixture of cross-subject and cross-protocol effects, which are difficult to separate. This could be a crucial disadvantage when establishing an imaging protocol that could minimize the cross-protocol effect in a multi-center study. On the other hand, when evaluating the robustness of image analysis tools with regard to the size of the biological effect, the separation of the cross-subject and cross-protocol contributions is not as crucial. This is especially true when the goal is to improve the robustness through image analysis algorithms and parameters using the same data sets.

The evaluation of the protocol difference on image analysis approaches is not straightforward. If an image analysis tool has an extremely low precision, the results would be dominated by noise, which could mask many kinds of effects, including the impact of protocol differences. Therefore, the statistical absence of the influence of different protocols does not necessarily mean a successful result. In this regard, the results, such as those depicted in Fig 2, need to be examined within the context of detection of biological effects. The granularity level of the structural definition is another important factor to consider. The smallest granularity we can achieve is, of course, a voxel. The voxel-based analysis is arguably the most widely used automated image analysis tool and there are a plethora of publications about this method. Operating on voxel units, the concept of granularity is relevant in VBA most likely only when the size of a spatial filter is in question. The multi-atlas approach is, however, inherently designed for image parcellation, and, thus, the granularity level of the structural definition is of central importance, and this is controlled by how structures are defined in the multiple atlases.

In our statistical analysis, the results with granularity levels 1–4 did not show any significant impact of the protocol differences. This makes sense because the structures in the lower granularity levels, such as the hemispheres, ventricles, the brainstem, and the cerebellum, can be demarcated with clear intensity differences and anatomical clues. Differences in contrasts and intensity homogeneity would influence the precision increasingly as the target structures become smaller and more local. In our analysis, there were two structures with significant differences among the six protocols at the highest granularity level. Because different subjects were scanned by the six different protocols, we cannot deny the possibility that the observed difference is due to biological effects (subject variability). However, these two structures, the inferior temporal lobe white matter (ITWM_L) and the right gyrus rectus (RGWM_R), are located in areas that are more severely affected by B0 susceptibility, and, thus, it is reasonable to assume that they are indeed due to protocol differences; some areas may be more affected by susceptibility than others, depending on the echo times, acquisition times, magnetic field strengths, and shimming functions.

It is possible that the protocol effects vary depending on the structural granularity levels. As described above, “statistically non-significant” could be due to robustness against protocol differences, as well as low precision levels of the tool. We assume that there is a general tendency that, as the granularity increases, the measurement precision decreases (more difficult to define smaller structural units reproducibly) and the protocol effect increases (greater impact of contrast differences for smaller structural units). Another issue is that the number of multiple comparisons for which to correct increases with the granularity level. It is important to realize that the lesser the sensitivity to protocol differences could also mean the lower the sensitivity to biological effects of interest. Therefore, it is essential to evaluate the protocol effects with respect to biological effects. In our analysis, we did not see significant impacts of protocol differences at granularity levels 1–4, while clear age effects could be observed. Of course, this is because the age effects are rather global, and, thus, the lower granularity analysis would be an ideal approach. If a given pathological condition is highly focal, the conclusion could be different. At level 5, the analysis of total variance (Fig 10) indicated that the age effect still dominates the variation compared to the protocol effects. This type of information, i.e., the amount of variation attributed to an effect of interest, is crucial for planning studies, because it allows calculation of the statistical power of the analysis. In this case, for instance, if the anatomical effect of the pathology is as large as the age effect, this approach should provide a high level of precision for delineating the pathology in question, but, if the pathological effect is much smaller, then the protocol difference would be problematic.

### Evaluation of pathology in AD patients

**Fig 11:**
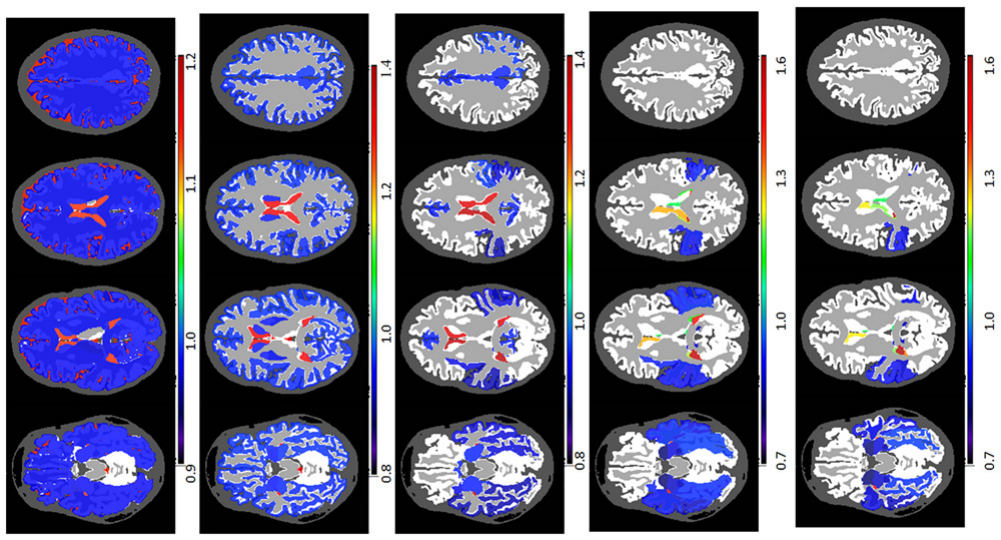
Differences in regional volumes between AD and controls at each granularity level. The colors code the ratio of volumes in AD/controls in regions of significant difference (P value<0.05, Bonferroni-corrected). Blue (ratio <1) represents regions of atrophy in AD, while green/yellow/red are regions that are bigger in AD, such as the ventricles.

In this study, we pooled the data from six different protocols and evaluated the pathological effect of AD, as a proof-of-concept. In general, AD patients frequently show visibly appreciable brain atrophy compared to age-matched healthy subjects. There are numerous publications about the brain atrophy patterns of AD patients using T1-weighted MRI, including the ADNI data. These include whole-brain atrophy and ventricle enlargement (see level 1 in Fig 11) [30–33], as well as atrophy of the temporal lobe (see level 3 in Fig 11)[34], the entorhinal cortex, and the hippocampus (see level 5 in Fig 11) [31,35–37]. McEvoy et al. [12] segmented the gray matter structures of ADNI data (1.5T, N = 84) and found the hippocampus, the entorhinal cortex, the middle and superior temporal gyri, the isthmus cingulate, and the orbital gyri the significant structures that discriminated AD patients. Our results agree with these findings, revealing atrophy in these areas, except for the isthmus cingulate and the orbital gyri, which had relatively minor contributions to the discrimination in McEvoy’s study. This suggests that the effect size of protocol differences is small enough to characterize the well-described anatomical features of AD patients.

## Conclusion

We describe the use of a fully automated image parcellation tool for brain MRI, using a multi-atlas approach. This performance may vary depending on the employed atlas sets and algorithms, as well as the degree of variability of the data protocols. Therefore, the exact values of the protocol effects reported in this paper are difficult to generalize. Nonetheless, our study provides a good estimation of what can be expected in terms of cross-protocol robustness for a fully automated image parcellation tool based on a multi-atlas probability-fusion algorithm. Namely, pathological effects on anatomy, which are evident in age-dependent or AD-caused atrophy, can be reliably detected within a reasonable level of variability for MPRAGE protocols. This result does not mean much in a conventional research environment, in which both patients and control subjects are scanned with the same scanning protocol. However, if automated quantification tools for clinical support are needed, a protocol difference could be a vital problem, because we cannot expect control data for every protocol used in hospitals. The robustness of the multi-atlas parcellation algorithm is an encouraging first step toward the use of existing medical images to improve our healthcare.

## Supporting Information

S1 Table Correlation coefficient(R) of age vs. normalized volumes. (DOCX)

S2 Table Mean and standard deviations of the normalized volumes in controls and AD individuals. (DOCX)

S3 Table Volumes of 832 regions of interest, into 5 levels of granularity, described in this study. (XLSX)
