# Supplementary material for: Evaluation of Cross-Protocol Stability of a Fully Automated Brain Multi-Atlas Parcellation Tool
Source: PLoS One. 2015 Jul 24;10(7):e0133533. doi: 10.1371/journal.pone.0133533 (PMC4514626; doi:10.1371/journal.pone.0133533)
Supplement: S1 Table — (DOCX) [file pone.0133533.s001.docx]

S1 Table

| Level1 | | | | | | | | | | | | | | | | | | |
| --- | --- | --- | --- | --- | --- | --- | --- | --- | --- | --- | --- | --- | --- | --- | --- | --- | --- | --- |
| Regions | | | | R-value | | | | Regions | | | R-value | | | Regions | | | R-value | |
| Telencephalon_L | | | | -0.61 | | | | Diencephalon_R | | | -0.34 | | | Myelencephalon | | | -0.02 | |
| Telencephalon_R | | | | -0.58 | | | | Mesencephalon | | | 0.24 | | | CSF | | | 0.70 | |
| Diencephalon_L | | | | -0.30 | | | | Metencephalon | | | 0.11 | | |  | | |  | |
| Level2 | | | | | | | | | | | | | | | | | | |
| Regions | | | R-value | | | | Regions | | | | R-value | | | | Regions | | R-value | |
| CerebralCortex_L | | | -0.53 | | | | BasalForebrain_R | | | | -0.24 | | | | WhiteMatter_L | | -0.44 | |
| CerebralCortex_R | | | -0.49 | | | | Mesencephalon_L | | | | -0.28 | | | | WhiteMatter_R | | -0.44 | |
| CerebralNuclei_L | | | -0.22 | | | | Mesencephalon_R | | | | -0.20 | | | | Ventricle | | 0.56 | |
| CerebralNuclei_R | | | -0.23 | | | | Metencephalon_R | | | | -0.11 | | | | Sulcus_L | | 0.61 | |
| Thalamus_L | | | -0.35 | | | | Metencephalon_L | | | | -0.12 | | | | Sulcus_R | | 0.63 | |
| Thalamus_R | | | -0.34 | | | | Myelencephalon_L | | | | -0.07 | | | |  | |  | |
| BasalForebrain_L | | | -0.12 | | | | Myelencephalon_R | | | | 0.02 | | | |  | |  | |
| Level3 | | | | | | | | | | | | | | | | | | |
| Regions | R-value | | | | Regions | | | | R-value | | | | Regions | | | | R-value | |
| Frontal lobe (left) | -0.47 | | | | Midbrain (left) | | | | -0.28 | | | | Lateral ventricle (left) | | | | 0.54 | |
| Frontal lobe (right) | -0.48 | | | | Midbrain (right) | | | | -0.20 | | | | Lateral ventricle (right) | | | | 0.55 | |
| patirtal lobe (left) | -0.34 | | | | cerebellum (right) | | | | -0.09 | | | | Third and fourth ventricles | | | | 0.55 | |
| parietal lobe (right) | -0.30 | | | | cerebellum (left) | | | | -0.11 | | | | sulci of the frontal lobe (left) | | | | 0.12 | |
| temporal lobe (left) | -0.39 | | | | pons (left) | | | | -0.18 | | | | sulci of the frontal lobe (right) | | | | 0.32 | |
| temporal lobe (right) | -0.38 | | | | pons (right) | | | | -0.20 | | | | Central sulcus (left) | | | | 0.46 | |
| limbic system (left) | -0.36 | | | | medulla (left) | | | | -0.07 | | | | Central sulcus (right) | | | | 0.50 | |
| limbic system (right) | -0.27 | | | | medulla (right) | | | | 0.02 | | | | Sylvian fissure and its extension (left) | | | | 0.61 | |
| Occipital lobe (left) | -0.04 | | | | Anterior part of the white matter (left) | | | | -0.49 | | | | Sylvian fissure and its extension (right) | | | | 0.56 | |
| Occipital lobe (right) | -0.01 | | | | Anterior part of the white matter (right) | | | | -0.55 | | | | sulci of the parietal lobe (left) | | | | 0.45 | |
| Insula (left) | -0.28 | | | | posterior part of the white matter (left) | | | | -0.17 | | | | sulci of the parietal lobe (right) | | | | 0.40 | |
| Insula (right) | -0.19 | | | | posterior part of the white matter (right) | | | | -0.14 | | | | sulci of the cingulate gyrus (left) | | | | 0.42 | |
| basal ganglia (left) | -0.14 | | | | Corpus callosum (left) | | | | 0.21 | | | | sulci of the cingulate gyrus (right) | | | | 0.44 | |
| basal ganglia (right) | -0.17 | | | | Corpus callosum (right) | | | | 0.18 | | | | sulci of the occipital lobe (left) | | | | 0.59 | |
| thalamus (left) | -0.35 | | | | Inferior part of the white matter (left) | | | | -0.32 | | | | sulci of the occipital lobe (right) | | | | 0.65 | |
| thalamus (right) | -0.34 | | | | Inferior part of the white matter (right) | | | | -0.39 | | | | Sulci of the temporal lobe (left) | | | | 0.54 | |
| Basal forebrain (left) | -0.12 | | | | White matter of the limbic system (left) | | | | -0.51 | | | | Sulci of the temporal lobe (right) | | | | 0.57 | |
| Basal forebrain (right) | -0.24 | | | | White matter of the limbic system (right) | | | | -0.39 | | | |  | | | |  | |
| Level4 | | | | | | | | | | | | | | | | | | |
| Regions | | | R-value | | | | Regions | | | | R-value | | Regions | | | | R-value | |
| Superior frontal gyrus (left) | | | -0.28 | | | | Caudate nucleus (left) | | | | 0.30 | | Fornix (left) | | | | 0.18 | |
| Superior frontal gyrus (right) | | | -0.29 | | | | Caudate nucleus (right) | | | | 0.22 | | Fornix (right) | | | | 0.20 | |
| Middle frontal gyrus (left) | | | -0.23 | | | | putamen (left) | | | | -0.37 | | peripheral parietal white matter (left) | | | | -0.24 | |
| Middle frontal gyrus (right) | | | -0.31 | | | | putamen (right) | | | | -0.35 | | peripheral parietal white matter (right) | | | | -0.27 | |
| Inferior frontal gyrus (left) | | | -0.25 | | | | Globus Pallidus (left) | | | | -0.22 | | Anterior part of the lateral ventricle (left) | | | | 0.50 | |
| Inferior frontal gyrus (right) | | | -0.19 | | | | Globus Pallidus (right) | | | | -0.20 | | posterior part of the lateral ventricle (left) | | | | 0.49 | |
| orbital gyrus (left) | | | -0.35 | | | | thalamus (left) | | | | -0.35 | | Inferior part of the lateral ventricle (left) | | | | 0.57 | |
| orbital gyrus (right) | | | -0.38 | | | | thalamus (right) | | | | -0.34 | | Anterior part of the lateral ventricle (right) | | | | 0.54 | |
| GYRUS RECTUS (left) | | | -0.47 | | | | Basal forebrain (left) | | | | -0.19 | | posterior part of the lateral ventricle (right) | | | | 0.52 | |
| GYRUS RECTUS (right) | | | -0.38 | | | | Basal forebrain (right) | | | | -0.34 | | Inferior part of the lateral ventricle (right) | | | | 0.48 | |
| Postcentral gyrus (left) | | | -0.29 | | | | midbrain (left) | | | | -0.28 | | Third and fourth ventricles | | | | 0.55 | |
| Postcentral gyrus (right) | | | -0.24 | | | | midbrain (right) | | | | -0.20 | | anterior part of the periventricular area (left) | | | | 0.49 | |
| Precentral gyrus (left) | | | -0.32 | | | | cerebellum (right) | | | | -0.09 | | anterior part of the periventricular area (right) | | | | 0.52 | |
| Precentral gyrus (right) | | | -0.30 | | | | cerebellum (left) | | | | -0.12 | | peripheral frontal white matter (left) | | | | -0.34 | |
| SUPERIOR PARIETAL LOBULE (left) | | | -0.16 | | | | pons (left) | | | | -0.18 | | peripheral frontal white matter (right) | | | | -0.44 | |
| SUPERIOR PARIETAL LOBULE (right) | | | -0.15 | | | | pons (right) | | | | -0.20 | | peripheral temporal white matter (left) | | | | -0.29 | |
| Supramarginal Gyrus (left) | | | -0.27 | | | | medulla (left) | | | | -0.07 | | peripheral temporal white matter (right) | | | | -0.38 | |
| Supramarginal Gyrus (right) | | | -0.16 | | | | medulla (right) | | | | 0.02 | | peripheral occipital white matter (left) | | | | 0.06 | |
| angular gyrus (left) | | | -0.09 | | | | anterior part of the Deep and periventricular white matter (left) | | | | -0.64 | | peripheral occipital white matter (right) | | | | 0.10 | |
| angular gyrus (right) | | | -0.23 | | | | anterior part of the Deep and periventricular white matter (right) | | | | -0.65 | | subcortical white matter of the cingulate gyrus (left) | | | | -0.47 | |
| Precuneus (left) | | | -0.16 | | | | posterior part of the Deep and periventricular white matter (left) | | | | -0.42 | | subcortical white matter of the cingulate gyrus (right) | | | | -0.33 | |
| Precuneus (right) | | | -0.17 | | | | posterior part of the Deep and periventricular white matter (right) | | | | -0.27 | | cerebellum white matter (right) | | | | -0.07 | |
| superior temporal gyrus (left) | | | -0.36 | | | | Genu of corpus callosum (left) | | | | 0.01 | | cerebellum white matter (left) | | | | -0.05 | |
| superior temporal gyrus (right) | | | -0.49 | | | | Genu of corpus callosum (right) | | | | -0.01 | | sulci of the frontal lobe (left) | | | | 0.12 | |
| Middle temporal gyrus (left) | | | -0.27 | | | | Body of corpus callosum (left) | | | | 0.33 | | sulci of the frontal lobe (right) | | | | 0.32 | |
| Middle temporal gyrus (right) | | | -0.17 | | | | Body of corpus callosum (right) | | | | 0.30 | | Central sulcus (left) | | | | 0.46 | |
| inferior temporal gyrus (left) | | | -0.24 | | | | Splenium of corpus callosum (left) | | | | 0.14 | | Central sulcus (right) | | | | 0.50 | |
| inferior temporal gyrus (right) | | | -0.16 | | | | Splenium of corpus callosum (right) | | | | 0.12 | | Sylvian fissure and anterior insular sulcus (left) | | | | 0.47 | |
| limbic system (left) | | | -0.11 | | | | posterior part of the periventricular area (left) | | | | 0.61 | | Sylvian fissure and anterior insular sulcus (right) | | | | 0.47 | |
| limbic system (right) | | | 0.06 | | | | posterior part of the periventricular area (right) | | | | 0.62 | | Sylvian fissure and posterior insular sulcus (left) | | | | 0.70 | |
| temporal lobe (left) | | | -0.27 | | | | Anterior limb of internal capsule (left) | | | | 0.05 | | Sylvian fissure and posterior insular sulcus (right) | | | | 0.60 | |
| temporal lobe (right) | | | -0.22 | | | | Anterior limb of internal capsule (right) | | | | 0.27 | | Extension of Sylvian fissure into supramarginal gyrus (left) | | | | 0.36 | |
| Occipital lobe (left) | | | -0.04 | | | | Posterior limb of internal capsule (left) | | | | -0.40 | | Extension of Sylvian fissure into supramarginal gyrus (right) | | | | 0.21 | |
| Occipital lobe (right) | | | -0.01 | | | | Posterior limb of internal capsule (right) | | | | -0.32 | | sulci of the parietal lobe (left) | | | | 0.45 | |
| Cingulate gyrus (left) | | | -0.33 | | | | inferior part of the Deep and periventricular white matter (left) | | | | -0.37 | | sulci of the parietal lobe (right) | | | | 0.40 | |
| Cingulate gyrus (right) | | | -0.25 | | | | inferior part of the Deep and periventricular white matter (right) | | | | -0.40 | | sulci of the cingulate gyrus (left) | | | | 0.42 | |
| Insula(left) | | | -0.28 | | | | Cingulum (cingulate gyrus part) (left) | | | | -0.37 | | sulci of the cingulate gyrus (right) | | | | 0.44 | |
| Insula(right) | | | -0.19 | | | | Cingulum (cingulate gyrus part) (right) | | | | -0.34 | | sulci of the occipital lobe (left) | | | | 0.59 | |
| Amygdala (left) | | | -0.32 | | | | Cingulum (hippocampal part) (left) | | | | -0.13 | | sulci of the occipital lobe (right) | | | | 0.65 | |
| Amygdala (right) | | | -0.30 | | | | Cingulum (hippocampal part) (right) | | | | -0.12 | | Sulci of the temporal lobe (left) | | | | 0.54 | |
| hippocampus (left) | | | -0.12 | | | | Fornix/stria terminalis (left) | | | | -0.34 | | Sulci of the temporal lobe (right) | | | | 0.57 | |
| hippocampus (right) | | | -0.26 | | | | Fornix/stria terminalis (right) | | | | -0.32 | |  | | | |  | |
| Level5 | | | | | | | | | | | | | | | | | | |
| Regions | | | R-value | | | | Regions | | | | R-value | | Regions | | | | R-value | |
| Superior frontal gyrus (left) | | | -0.32 | | | | red nucleus (left) | | | | -0.34 | | subcortical white matter of the superior frontal gyrus/ pole (right) | | | | 0.05 | |
| Superior frontal gyrus (right) | | | -0.14 | | | | red nucleus (right) | | | | -0.14 | | subcortical white matter of the middle frontal gyrus (left) | | | | -0.22 | |
| superior frontal gyrus/ prefrontal cortex (left) | | | -0.15 | | | | substantia nigra (left) | | | | -0.41 | | subcortical white matter of the middle frontal gyrus (right) | | | | -0.37 | |
| superior frontal gyrus/ prefrontal cortex (right) | | | -0.33 | | | | substantia nigra (right) | | | | -0.49 | | subcortical white matter of the middle frontal gyrus/ dorsolateral prefrontal cortex (left) | | | | -0.12 | |
| superior frontal gyrus/ pole (left) | | | 0.14 | | | | cerebellum gray matter (right) | | | | -0.09 | | subcortical white matter of the middle frontal gyrus/ dorsolateral prefrontal cortex (right) | | | | -0.32 | |
| superior frontal gyru/ pole (right) | | | -0.05 | | | | cerebellum gray matter (left) | | | | -0.12 | | subcortical white matter of the inferior frontal gyrus/pars opecularis (left) | | | | -0.31 | |
| Middle frontal gyrus (left) | | | -0.19 | | | | Cerebral peduncle (left) | | | | -0.34 | | subcortical white matter of the inferior frontal gyrus/pars opecularis (right) | | | | -0.31 | |
| Middle frontal gyrus (right) | | | -0.14 | | | | Cerebral peduncle (right) | | | | -0.21 | | subcortical white matter of the inferior frontal gyrus/pars orbitalis (left) | | | | -0.12 | |
| Middle frontal gyrus (dorsolateral prefrontal cortex) (left) | | | -0.19 | | | | midbrain (left) | | | | -0.04 | | subcortical white matter of the inferior frontal gyrus/pars orbitalis (right) | | | | -0.25 | |
| Middle frontal gyrus (dorsolateral prefrontal cortex) (right) | | | -0.32 | | | | midbrain (right) | | | | -0.06 | | subcortical white matter of the inferior frontal gyrus/pars triangularis (left) | | | | -0.32 | |
| inferior frontal gyrus/pars opercularis (left) | | | -0.38 | | | | corticospinal tract (left) | | | | -0.09 | | subcortical white matter of the inferior frontal gyrus/pars triangularis (right) | | | | -0.22 | |
| inferior frontal gyrus/pars opercularis (right) | | | -0.14 | | | | corticospinal tract (right) | | | | -0.14 | | subcortical white matter of the lateral fronto-orbital gyrus (left) | | | | -0.14 | |
| inferior frontal gyrus/pars orbitalis (left) | | | -0.15 | | | | superior cerebellar peduncle (left) | | | | -0.16 | | subcortical white matter of the lateral fronto-orbital gyrus (right) | | | | -0.22 | |
| inferior frontal gyrus/pars orbitalis (right) | | | -0.26 | | | | superior cerebellar peduncle (right) | | | | -0.31 | | subcortical white matter of the middle fronto-orbital gyrus (left) | | | | -0.05 | |
| inferior frontal gyrus/ pars triangularis (left) | | | -0.09 | | | | middle cerebellar peduncle (left) | | | | -0.12 | | subcortical white matter of the middle fronto-orbital gyrus (right) | | | | -0.01 | |
| inferior frontal gyrus/ pars triangularis (right) | | | -0.06 | | | | middle cerebellar peduncle (right) | | | | -0.14 | | subcortical white matter of the gyrus rectus (left) | | | | -0.05 | |
| LATERAL FRONTO-ORBITAL GYRUS (left) | | | -0.24 | | | | Pontine crossing tract (left) | | | | -0.16 | | subcortical white matter of the gyrus rectus (right) | | | | -0.06 | |
| LATERAL FRONTO-ORBITAL GYRUS (right) | | | -0.33 | | | | Pontine crossing tract (right) | | | | -0.27 | | subcortical white matter of the postcentral gyrus (left) | | | | 0.01 | |
| MIDDLE FRONTO-ORBITAL GYRUS (left) | | | -0.34 | | | | Inferior cerebellar peduncle (left) | | | | -0.07 | | subcortical white matter of the postcentral gyrus (right) | | | | 0.09 | |
| MIDDLE FRONTO-ORBITAL GYRUS (right) | | | -0.29 | | | | Inferior cerebellar peduncle (right) | | | | 0.01 | | subcortical white matter of the precentral gyrus (left) | | | | -0.12 | |
| GYRUS RECTUS (left) | | | -0.47 | | | | medial lemniscus (left) | | | | -0.31 | | subcortical white matter of the precentral gyrus (right) | | | | -0.09 | |
| GYRUS RECTUS (right) | | | -0.38 | | | | medial lemniscus (right) | | | | -0.15 | | subcortical white matter of the superior parietal lobule (left) | | | | -0.22 | |
| Postcentral gyrus (left) | | | -0.29 | | | | pons (left) | | | | -0.06 | | subcortical white matter of the superior parietal lobule (right) | | | | -0.29 | |
| Postcentral gyrus (right) | | | -0.24 | | | | pons (right) | | | | -0.04 | | subcortical white matter of the supramarginal gyrus (left) | | | | -0.06 | |
| Precentral gyrus (left) | | | -0.32 | | | | medulla (left) | | | | -0.06 | | subcortical white matter of the supramarginal gyrus (right) | | | | 0.03 | |
| Precentral gyrus (right) | | | -0.30 | | | | medulla (right) | | | | 0.03 | | Angular white matter (left) | | | | 0.28 | |
| SUPERIOR PARIETAL LOBULE (left) | | | -0.16 | | | | Anterior corona radiata (left) | | | | -0.56 | | Anterior white matter (right) | | | | 0.05 | |
| SUPERIOR PARIETAL LOBULE (right) | | | -0.15 | | | | Anterior corona radiata (right) | | | | -0.64 | | subcortical white matter of the precuneus (left) | | | | 0.18 | |
| Supramarginal Gyrus (left) | | | -0.27 | | | | Superior corona radiata (left) | | | | -0.57 | | subcortical white matter of the precuneus (right) | | | | 0.28 | |
| Supramarginal Gyrus (right) | | | -0.16 | | | | Superior corona radiata (right) | | | | -0.52 | | subcortical white matter of the superior temporal gyrus (left) | | | | -0.20 | |
| angular gyrus (left) | | | -0.09 | | | | Posterior corona radiata (left) | | | | -0.42 | | subcortical white matter of the superior temporal gyrus (right) | | | | -0.44 | |
| angular gyrus (right) | | | -0.23 | | | | Posterior corona radiata (right) | | | | -0.27 | | subcortical white matter of the superior temporal gyrus/ pole (left) | | | | 0.36 | |
| Precuneus (left) | | | -0.16 | | | | Genu of corpus callosum (left) | | | | 0.01 | | subcortical white matter of the superior temporal gyrus/ pole (right) | | | | 0.35 | |
| Precuneus (right) | | | -0.17 | | | | Genu of corpus callosum (right) | | | | -0.01 | | subcortical white matter of the middle temporal gyrus (left) | | | | -0.54 | |
| superior temporal gyrus (left) | | | -0.30 | | | | Body of corpus callosum (left) | | | | 0.33 | | subcortical white matter of the middle temporal gyrus (right) | | | | -0.36 | |
| superior temporal gyrus (right) | | | -0.46 | | | | Body of corpus callosum (right) | | | | 0.30 | | subcortical white matter of the middle temporal gyrus/ pole (left) | | | | 0.19 | |
| superior temporal gyrus/ pole (left) | | | -0.30 | | | | Splenium of corpus callosum (left) | | | | 0.14 | | subcortical white matter of the middle temporal gyrus/ pole (right) | | | | 0.29 | |
| superior temporal gyrus/ pole (right) | | | -0.28 | | | | Splenium of corpus callosum (right) | | | | 0.12 | | subcortical white matter of the inferior temporal gyrus (left) | | | | 0.03 | |
| Middle temporal gyrus (left) | | | -0.25 | | | | lateral part of the periventricular white matter (left) | | | | 0.55 | | subcortical white matter of the inferior temporal gyrus (right) | | | | -0.04 | |
| Middle temporal gyrus (right) | | | -0.18 | | | | lateral part of the periventricular white matter (right) | | | | 0.54 | | subcortical white matter of the fusiform gyrus (left) | | | | -0.24 | |
| Middle temporal gyrus_pole (left) | | | -0.09 | | | | Anterior limb of internal capsule (left) | | | | 0.05 | | subcortical white matter of the fusiform gyrus (right) | | | | -0.05 | |
| Middle temporal gyrus_pole (right) | | | -0.00 | | | | Anterior limb of internal capsule (right) | | | | 0.27 | | subcortical white matter of the superior occipital gyrus (left) | | | | -0.13 | |
| inferior temporal gyrus (left) | | | -0.24 | | | | Posterior limb of internal capsule (left) | | | | -0.40 | | subcortical white matter of the superior occipital gyrus (right) | | | | 0.05 | |
| inferior temporal gyrus (right) | | | -0.16 | | | | Posterior limb of internal capsule (right) | | | | -0.32 | | subcortical white matter of the middle occiptial gyrus (left) | | | | -0.09 | |
| Parahippocampal gyrus (left) | | | -0.20 | | | | retrolenticular part of internal capsule (left) | | | | -0.42 | | subcortical white matter of the middle occiptial gyrus (right) | | | | 0.04 | |
| Parahippocampal gyrus (right) | | | 0.04 | | | | retrolenticular part of internal capsule (right) | | | | -0.24 | | subcortical wihte matter of the inferior occipital gyrus (left) | | | | -0.15 | |
| ENTORHINAL AREA (left) | | | 0.04 | | | | external capsule (left) | | | | -0.14 | | subcortical white matter of the inferior occipital gyrus (right) | | | | -0.22 | |
| ENTORHINAL AREA (right) | | | 0.07 | | | | external capsule (right) | | | | -0.21 | | subcortical white matter of the cuneus (left) | | | | 0.35 | |
| Fusiform gyrus (left) | | | -0.27 | | | | Cingulum (cingulate gyrus part) (left) | | | | -0.37 | | subcortical white matter of the cuneus (right) | | | | 0.29 | |
| Fusiform gyrus (right) | | | -0.22 | | | | Cingulum (cingulate gyrus part) (right) | | | | -0.34 | | subcortical white matter of the lingual gyrus (left) | | | | 0.16 | |
| SUPERIOR OCCIPITAL GYRUS (left) | | | -0.09 | | | | Cingulum (hippocampal part) (left) | | | | -0.13 | | subcortical white matter of the lingual gyrus (right) | | | | 0.10 | |
| SUPERIOR OCCIPITAL GYRUS (right) | | | -0.02 | | | | Cingulum (hippocampal part) (right) | | | | -0.12 | | subcortical white matter of the rostral anterior cingulate cortex (left) | | | | -0.02 | |
| middle occipital gyrus (left) | | | -0.20 | | | | Fornix/stria terminalis (left) | | | | -0.34 | | subcortical white matter of the rostral anterior cingulate cortex (right) | | | | -0.08 | |
| middle occiptial gyrus (right) | | | -0.09 | | | | Fornix/stria terminalis (right) | | | | -0.32 | | subcortical white matter of the subcallosal anterior cingulate cortex (left) | | | | -0.31 | |
| inferior occipital gyrus (left) | | | -0.21 | | | | Fornix (left) | | | | 0.18 | | subcortical white matter of the subcallosal anterior cingulate cortex (right) | | | | -0.03 | |
| inferior occipital gyrus (right) | | | -0.05 | | | | Fornix (right) | | | | 0.20 | | subcortical white matter of the subgenual anterior cingulate cortex (left) | | | | 0.11 | |
| Cuneus (left) | | | 0.12 | | | | Inferior fronto-occipital fasciculus (left) | | | | 0.10 | | subcortical white matter of the subgenual anterior cingulate cortex (right) | | | | 0.06 | |
| Cuneus (right) | | | 0.09 | | | | Inferior fronto-occipital fasciculus (right) | | | | -0.11 | | subcortical white matter of the dorsal anterior cingulate cortex (left) | | | | -0.26 | |
| Lingual gyrus (left) | | | 0.39 | | | | Posterior thalamic radiation (left) | | | | -0.29 | | subcortical white matter of the dorsal anterior cingulate cortex (right) | | | | -0.27 | |
| Lingual gyrus (right) | | | 0.18 | | | | Posterior thalamic radiation (right) | | | | -0.20 | | subcortical white matter of the postrior cingulate cortex (left) | | | | -0.39 | |
| rostral_Anterior cingulate cortex (left) | | | -0.27 | | | | Sagittal stratum (left) | | | | -0.33 | | subcortical white matter of the posterior cingulate cortex (right) | | | | -0.26 | |
| rostral_Anterior cingulate cortex (right) | | | -0.03 | | | | Sagittal stratum (right) | | | | -0.53 | | cerebellum white matter (right) | | | | -0.02 | |
| subcallosal_Anterior cingulate cortex (left) | | | -0.09 | | | | Superior fronto-occipital fascicul (left) | | | | 0.07 | | cerebellum white matter (left) | | | | 0.02 | |
| subcallosal_Anterior cingulate cortex (right) | | | -0.26 | | | | Superior fronto-occipital fascicul (right) | | | | 0.24 | | middle cerebellar peduncle in the cerebellum (left) | | | | -0.21 | |
| subgenual anterior cingulate cortex (left) | | | -0.22 | | | | Superior longitudinal fasciculus (left) | | | | -0.61 | | middle cerebellar peduncle in the cerebellum (right) | | | | -0.21 | |
| subgenual anterior cingulate cortex (right) | | | -0.07 | | | | Superior longitudinal fasciculus (right) | | | | -0.56 | | Inferior cerebellar peduncle/pons (left) | | | | -0.34 | |
| dorsal Anterior cingulate cortex (left) | | | -0.36 | | | | claustrum (left) | | | | -0.06 | | Inferior cerebellar peduncle/pons (right) | | | | -0.20 | |
| dorsal Anterior cingulate cortex (right) | | | -0.37 | | | | claustrum (right) | | | | -0.43 | | sulci of the frontal lobe (left) | | | | 0.12 | |
| Posterior cingulate cortex (left) | | | -0.06 | | | | Mammillary body (right) | | | | 0.07 | | sulci of the frontal lobe (right) | | | | 0.32 | |
| Posterior cingulate cortex (right) | | | -0.03 | | | | Mammillary body (left) | | | | -0.01 | | Central sulcus (left) | | | | 0.46 | |
| Insula (left) | | | -0.28 | | | | frontal horn of the lateral ventricle (left) | | | | 0.50 | | Central sulcus (right) | | | | 0.50 | |
| Insula (right) | | | -0.19 | | | | body of the lateral ventricle (left) | | | | 0.47 | | Sylvian fissure and anterior insular sulcus (left) | | | | 0.47 | |
| Amygdala (left) | | | -0.32 | | | | Lateral ventricle, atrium part (left) | | | | 0.47 | | Sylvian fissure and anterior insular sulcus (right) | | | | 0.47 | |
| Amygdala (right) | | | -0.30 | | | | Occipital horn of the lateral ventricle (left) | | | | 0.42 | | Sylvian fissure and posterior insular sulcus (left) | | | | 0.70 | |
| hippocampus (left) | | | -0.12 | | | | inferior horn of the lateral ventricle (left) | | | | 0.57 | | Sylvian fissure and posterior insular sulcus (right) | | | | 0.60 | |
| hippocampus (right) | | | -0.26 | | | | frontal horn of the lateral ventricle (right) | | | | 0.52 | | Extension of Sylvian fissure into supramarginal gyrus (left) | | | | 0.36 | |
| Caudate nucleus (left) | | | 0.30 | | | | body of the lateral ventricle (right) | | | | 0.52 | | Extension of Sylvian fissure into supramarginal gyrus (right) | | | | 0.21 | |
| Caudate nucleus (right) | | | 0.22 | | | | Lateral ventricle, atrium part (right) | | | | 0.52 | | sulci of the parietal lobe (left) | | | | 0.45 | |
| putamen (left) | | | -0.37 | | | | Occipital horn of the lateral ventricle (right) | | | | 0.39 | | sulci of the parietal lobe (right) | | | | 0.40 | |
| putamen (right) | | | -0.35 | | | | inferior horn of the lateral ventricle (right) | | | | 0.48 | | sulci of the cingulate gyrus (left) | | | | 0.42 | |
| Globus Pallidus (left) | | | -0.22 | | | | Third and fourth ventricles | | | | 0.54 | | sulci of the cingulate gyrus (right) | | | | 0.44 | |
| Globus Pallidus (right) | | | -0.20 | | | | anterior part of the periventricular white matter (left) | | | | 0.48 | | sulci of the occipital lobe (left) | | | | 0.59 | |
| thalamus (left) | | | -0.35 | | | | anterior part of the periventricular white matter (right) | | | | 0.52 | | sulci of the occipital lobe (right) | | | | 0.65 | |
| thalamus (right) | | | -0.34 | | | | posterior part of the periventricular white matter (left) | | | | 0.56 | | Sulci of the temporal lobe (left) | | | | 0.54 | |
| Hypothalamus (left) | | | 0.01 | | | | posterior part of the periventricular white matter (right) | | | | 0.66 | | Sulci of the temporal lobe (right) | | | | 0.57 | |
| Hypothalamus (right) | | | -0.09 | | | | subcortical white matter of the superior frontal gyrus (left) | | | | -0.23 | | Fimbria (left) | | | | 0.04 | |
| Basal forebrain (left) | | | -0.12 | | | | subcortical white matter of the superior frontal gyrus (right) | | | | -0.17 | | Fimbria (right) | | | | -0.01 | |
| Basal forebrain (right) | | | -0.02 | | | | subcortical white matter of the superior frontal gyrus/ prefrontal cortex (left) | | | | -0.27 | | Choroid plexus of the lateral ventricle etc (left) | | | | 0.29 | |
| Nucleus accumbens (left) | | | -0.33 | | | | subcortical white matter of the superior frontal gyrus/ prefrontal cortex (right) | | | | -0.37 | | Choroid plexus of the lateral ventricle etc (right) | | | | 0.41 | |
| Nucleus accumbens (right) | | | -0.45 | | | | subcortical white matter of the superior frontal gyrus/ pole (left) | | | | 0.13 | |  | | | |  | |
