# Supplementary material for: Evaluation of Cross-Protocol Stability of a Fully Automated Brain Multi-Atlas Parcellation Tool
Source: PLoS One. 2015 Jul 24;10(7):e0133533. doi: 10.1371/journal.pone.0133533 (PMC4514626; doi:10.1371/journal.pone.0133533)
Supplement: S2 Table — (DOCX) [file pone.0133533.s002.docx]

S2 Table

|  | Control | | | | | | AD | | |
| --- | --- | --- | --- | --- | --- | --- | --- | --- | --- |
| Regions | mean | | | std | | | mean | | std |
| Level1 | | | | | | | | | |
| Telencephalon_L | 0.370819 | | | 0.012274 | | | 0.361615 | | 0.012643 |
| Telencephalon_R | 0.370463 | | | 0.012695 | | | 0.36018 | | 0.01093 |
| Diencephalon_L | 0.006209 | | | 0.000453 | | | 0.00604 | | 0.000425 |
| Diencephalon_R | 0.006162 | | | 0.00046 | | | 0.005954 | | 0.000416 |
| Mesencephalon | 0.007738 | | | 0.000593 | | | 0.007486 | | 0.000626 |
| Metencephalon | 0.107678 | | | 0.012164 | | | 0.109679 | | 0.00899 |
| Myelencephalon | 0.003556 | | | 0.000672 | | | 0.003626 | | 0.000482 |
| CSF | 0.127373 | | | 0.023997 | | | 0.14542 | | 0.023662 |
| Level2 | | | | | | | | | |
| CerebralCortex_L | 0.192856 | | | 0.007877 | | | 0.186432 | | 0.00753 |
| CerebralCortex_R | 0.192743 | | | 0.008238 | | | 0.185301 | | 0.006782 |
| CerebralNuclei_L | 0.008452 | | | 0.000543 | | | 0.00804 | | 0.000699 |
| CerebralNuclei_R | 0.008992 | | | 0.000656 | | | 0.008562 | | 0.000732 |
| Thalamus_L | 0.003953 | | | 0.000331 | | | 0.003877 | | 0.000316 |
| Thalamus_R | 0.004007 | | | 0.00036 | | | 0.003895 | | 0.000323 |
| BasalForebrain_L | 0.002257 | | | 0.000167 | | | 0.002164 | | 0.000178 |
| BasalForebrain_R | 0.002155 | | | 0.000155 | | | 0.00206 | | 0.00018 |
| Mesencephalon_L | 0.003923 | | | 0.000307 | | | 0.003801 | | 0.000327 |
| Mesencephalon_R | 0.003816 | | | 0.000299 | | | 0.003686 | | 0.000315 |
| Metencephalon_R | 0.053762 | | | 0.006482 | | | 0.054638 | | 0.005193 |
| Metencephalon_L | 0.053917 | | | 0.005974 | | | 0.055041 | | 0.004202 |
| Myelencephalon_L | 0.001701 | | | 0.000327 | | | 0.001754 | | 0.000217 |
| Myelencephalon_R | 0.001855 | | | 0.000361 | | | 0.001872 | | 0.000276 |
| WhiteMatter_L | 0.169513 | | | 0.00731 | | | 0.167144 | | 0.007386 |
| WhiteMatter_R | 0.168729 | | | 0.007283 | | | 0.166317 | | 0.006435 |
| Ventricle | 0.035112 | | | 0.013776 | | | 0.046415 | | 0.016966 |
| Sulcus_L | 0.047882 | | | 0.007147 | | | 0.051229 | | 0.00747 |
| Sulcus_R | 0.04438 | | | 0.007269 | | | 0.047777 | | 0.006412 |
| Level3 | | | | | | | | | |
| Frontal lobe (left) | 0.061849 | | | 0.003447 | | | 0.060742 | | 0.003504 |
| Frontal lobe (right) | 0.060917 | | | 0.003442 | | | 0.059514 | | 0.003519 |
| patirtal lobe (left) | 0.031812 | | | 0.00239 | | | 0.030798 | | 0.002319 |
| parietal lobe (right) | 0.034263 | | | 0.002491 | | | 0.033476 | | 0.002796 |
| temporal lobe (left) | 0.046332 | | | 0.002762 | | | 0.04317 | | 0.003266 |
| temporal lobe (right) | 0.044198 | | | 0.002989 | | | 0.040287 | | 0.00382 |
| limbic system (left) | 0.021099 | | | 0.001232 | | | 0.020249 | | 0.001225 |
| limbic system (right) | 0.022033 | | | 0.0014 | | | 0.021108 | | 0.0013 |
| Occipital lobe (left) | 0.028196 | | | 0.002132 | | | 0.028008 | | 0.002545 |
| Occipital lobe (right) | 0.027525 | | | 0.002253 | | | 0.027205 | | 0.002721 |
| Insula (left) | 0.004783 | | | 0.000484 | | | 0.004468 | | 0.000479 |
| Insula (right) | 0.005142 | | | 0.000508 | | | 0.004809 | | 0.000437 |
| basal ganglia (left) | 0.007238 | | | 0.000517 | | | 0.007039 | | 0.000644 |
| basal ganglia (right) | 0.007659 | | | 0.000628 | | | 0.007466 | | 0.000679 |
| thalamus (left) | 0.003953 | | | 0.000331 | | | 0.003877 | | 0.000316 |
| thalamus (right) | 0.004007 | | | 0.00036 | | | 0.003895 | | 0.000323 |
| Basal forebrain (left) | 0.002257 | | | 0.000167 | | | 0.002164 | | 0.000178 |
| Basal forebrain (right) | 0.002155 | | | 0.000155 | | | 0.00206 | | 0.00018 |
| Midbrain (left) | 0.003923 | | | 0.000307 | | | 0.003801 | | 0.000327 |
| Midbrain (right) | 0.003816 | | | 0.000299 | | | 0.003686 | | 0.000315 |
| cerebellum (right) | 0.046701 | | | 0.006177 | | | 0.04781 | | 0.004867 |
| cerebellum (left) | 0.04757 | | | 0.005612 | | | 0.048861 | | 0.003871 |
| pons (left) | 0.006348 | | | 0.000668 | | | 0.00618 | | 0.000706 |
| pons (right) | 0.007061 | | | 0.000759 | | | 0.006828 | | 0.000764 |
| medulla (left) | 0.001701 | | | 0.000327 | | | 0.001754 | | 0.000217 |
| medulla (right) | 0.001855 | | | 0.000361 | | | 0.001872 | | 0.000276 |
| Anterior part of the white matter (left) | 0.059224 | | | 0.004091 | | | 0.058411 | | 0.004964 |
| Anterior part of the white matter (right) | 0.059169 | | | 0.00373 | | | 0.058586 | | 0.004589 |
| posterior part of the white matter (left) | 0.051696 | | | 0.003183 | | | 0.051458 | | 0.002609 |
| posterior part of the white matter (right) | 0.051746 | | | 0.003298 | | | 0.051262 | | 0.003016 |
| Corpus callosum (left) | 0.009792 | | | 0.000962 | | | 0.009918 | | 0.001072 |
| Corpus callosum (right) | 0.010259 | | | 0.00105 | | | 0.01052 | | 0.001004 |
| Inferior part of the white matter (left) | 0.040475 | | | 0.001683 | | | 0.039224 | | 0.002098 |
| Inferior part of the white matter (right) | 0.03949 | | | 0.001747 | | | 0.038055 | | 0.002136 |
| White matter of the limbic system (left) | 0.008327 | | | 0.000594 | | | 0.008134 | | 0.000525 |
| White matter of the limbic system (right) | 0.008066 | | | 0.000544 | | | 0.007895 | | 0.000585 |
| Lateral ventricle (left) | 0.01517 | | | 0.006392 | | | 0.020343 | | 0.008703 |
| Lateral ventricle (right) | 0.015748 | | | 0.006979 | | | 0.021694 | | 0.008385 |
| Third and fourth ventricles | 0.004194 | | | 0.000834 | | | 0.004378 | | 0.000915 |
| sulci of the frontal lobe (left) | 0.02006 | | | 0.00267 | | | 0.021319 | | 0.002812 |
| sulci of the frontal lobe (right) | 0.016161 | | | 0.002298 | | | 0.01709 | | 0.002159 |
| Central sulcus (left) | 0.00276 | | | 0.000711 | | | 0.002804 | | 0.00075 |
| Central sulcus (right) | 0.002762 | | | 0.000722 | | | 0.00288 | | 0.00067 |
| Sylvian fissure and its extension (left) | 0.006788 | | | 0.001989 | | | 0.008072 | | 0.002543 |
| Sylvian fissure and its extension (right) | 0.007109 | | | 0.001866 | | | 0.008213 | | 0.002015 |
| sulci of the parietal lobe (left) | 0.008602 | | | 0.002017 | | | 0.008627 | | 0.001787 |
| sulci of the parietal lobe (right) | 0.00931 | | | 0.002187 | | | 0.0095 | | 0.001841 |
| sulci of the cingulate gyrus (left) | 0.004162 | | | 0.001142 | | | 0.00432 | | 0.001138 |
| sulci of the cingulate gyrus (right) | 0.003774 | | | 0.001045 | | | 0.004106 | | 0.001109 |
| sulci of the occipital lobe (left) | 0.003168 | | | 0.00119 | | | 0.003343 | | 0.001048 |
| sulci of the occipital lobe (right) | 0.003403 | | | 0.001168 | | | 0.003773 | | 0.001134 |
| Sulci of the temporal lobe (left) | 0.002344 | | | 0.000765 | | | 0.002746 | | 0.000611 |
| Sulci of the temporal lobe (right) | 0.001862 | | | 0.000644 | | | 0.002218 | | 0.000565 |
| Level4 | | | | | | | | | |
| Superior frontal gyrus (left) | | | 0.017763 | 0.001332 | | | 0.017367 | | 0.001538 |
| Superior frontal gyrus (right) | | | 0.016434 | 0.00125 | | | 0.015878 | | 0.001389 |
| Middle frontal gyrus (left) | | | 0.016035 | 0.00142 | | | 0.01542 | | 0.001439 |
| Middle frontal gyrus (right) | | | 0.016269 | 0.001563 | | | 0.015671 | | 0.001275 |
| Inferior frontal gyrus (left) | | | 0.007728 | 0.000841 | | | 0.007521 | | 0.000768 |
| Inferior frontal gyrus (right) | | | 0.007001 | 0.00078 | | | 0.006885 | | 0.000708 |
| orbital gyrus (left) | | | 0.006883 | 0.00056 | | | 0.006778 | | 0.0006 |
| orbital gyrus (right) | | | 0.006462 | 0.000539 | | | 0.006236 | | 0.000558 |
| GYRUS RECTUS (left) | | | 0.003717 | 0.000421 | | | 0.003603 | | 0.00048 |
| GYRUS RECTUS (right) | | | 0.003667 | 0.000384 | | | 0.003569 | | 0.000512 |
| Postcentral gyrus (left) | | | 0.008347 | 0.000931 | | | 0.008184 | | 0.000809 |
| Postcentral gyrus (right) | | | 0.008412 | 0.000768 | | | 0.008479 | | 0.000782 |
| Precentral gyrus (left) | | | 0.009724 | 0.001014 | | | 0.010055 | | 0.001075 |
| Precentral gyrus (right) | | | 0.011088 | 0.0011 | | | 0.011276 | | 0.000946 |
| SUPERIOR PARIETAL LOBULE (left) | | | 0.006386 | 0.000942 | | | 0.006337 | | 0.000956 |
| SUPERIOR PARIETAL LOBULE (right) | | | 0.005509 | 0.000964 | | | 0.005315 | | 0.001135 |
| Supramarginal Gyrus (left) | | | 0.006537 | 0.00098 | | | 0.006244 | | 0.000888 |
| Supramarginal Gyrus (right) | | | 0.006829 | 0.000732 | | | 0.00672 | | 0.000788 |
| angular gyrus (left) | | | 0.005543 | 0.000638 | | | 0.005239 | | 0.000664 |
| angular gyrus (right) | | | 0.008901 | 0.000906 | | | 0.00862 | | 0.001004 |
| Precuneus (left) | | | 0.005001 | 0.000522 | | | 0.004796 | | 0.000492 |
| Precuneus (right) | | | 0.004614 | 0.000539 | | | 0.004343 | | 0.000658 |
| superior temporal gyrus (left) | | | 0.012403 | 0.00108 | | | 0.011479 | | 0.001044 |
| superior temporal gyrus (right) | | | 0.012057 | 0.001194 | | | 0.010999 | | 0.001296 |
| Middle temporal gyrus (left) | | | 0.013896 | 0.001087 | | | 0.013 | | 0.00136 |
| Middle temporal gyrus (right) | | | 0.011433 | 0.001247 | | | 0.010348 | | 0.001254 |
| inferior temporal gyrus (left) | | | 0.008885 | 0.000863 | | | 0.00803 | | 0.000874 |
| inferior temporal gyrus (right) | | | 0.009735 | 0.000982 | | | 0.008561 | | 0.001257 |
| limbic system (left) | | | 0.001567 | 0.000153 | | | 0.001405 | | 0.000208 |
| limbic system (right) | | | 0.001821 | 0.000202 | | | 0.001648 | | 0.000298 |
| temporal lobe (left) | | | 0.01115 | 0.000724 | | | 0.010662 | | 0.000818 |
| temporal lobe (right) | | | 0.010974 | 0.000799 | | | 0.010381 | | 0.001054 |
| Occipital lobe (left) | | | 0.028196 | 0.002132 | | | 0.028008 | | 0.002545 |
| Occipital lobe (right) | | | 0.027525 | 0.002253 | | | 0.027205 | | 0.002721 |
| Cingulate gyrus (left) | | | 0.01574 | 0.001089 | | | 0.015519 | | 0.000991 |
| Cingulate gyrus (right) | | | 0.016182 | 0.001154 | | | 0.015985 | | 0.000982 |
| Insula(left) | | | 0.004783 | 0.000484 | | | 0.004468 | | 0.000479 |
| Insula(right) | | | 0.005142 | 0.000508 | | | 0.004809 | | 0.000437 |
| Amygdala (left) | | | 0.001214 | 0.000143 | | | 0.001002 | | 0.000146 |
| Amygdala (right) | | | 0.001334 | 0.000145 | | | 0.001097 | | 0.000159 |
| hippocampus (left) | | | 0.002579 | 0.00024 | | | 0.002325 | | 0.000285 |
| hippocampus (right) | | | 0.002698 | 0.000261 | | | 0.00238 | | 0.000306 |
| Caudate nucleus (left) | | | 0.002773 | 0.00027 | | | 0.002768 | | 0.000338 |
| Caudate nucleus (right) | | | 0.002804 | 0.000311 | | | 0.002806 | | 0.000366 |
| putamen (left) | | | 0.00296 | 0.000295 | | | 0.002796 | | 0.000381 |
| putamen (right) | | | 0.003146 | 0.000338 | | | 0.002975 | | 0.000391 |
| Globus Pallidus (left) | | | 0.001064 | 0.000125 | | | 0.001061 | | 0.000117 |
| Globus Pallidus (right) | | | 0.001141 | 0.000128 | | | 0.001141 | | 0.000137 |
| thalamus (left) | | | 0.003953 | 0.000331 | | | 0.003877 | | 0.000316 |
| thalamus (right) | | | 0.004007 | 0.00036 | | | 0.003895 | | 0.000323 |
| Basal forebrain (left) | | | 0.0027 | 0.000201 | | | 0.002578 | | 0.000205 |
| Basal forebrain (right) | | | 0.002723 | 0.000197 | | | 0.002606 | | 0.00022 |
| midbrain (left) | | | 0.003923 | 0.000307 | | | 0.003801 | | 0.000327 |
| midbrain (right) | | | 0.003816 | 0.000299 | | | 0.003686 | | 0.000315 |
| cerebellum (right) | | | 0.037611 | 0.004981 | | | 0.038365 | | 0.00404 |
| cerebellum (left) | | | 0.038307 | 0.004518 | | | 0.039282 | | 0.003273 |
| pons (left) | | | 0.006348 | 0.000668 | | | 0.00618 | | 0.000706 |
| pons (right) | | | 0.007061 | 0.000759 | | | 0.006828 | | 0.000764 |
| medulla (left) | | | 0.001701 | 0.000327 | | | 0.001754 | | 0.000217 |
| medulla (right) | | | 0.001855 | 0.000361 | | | 0.001872 | | 0.000276 |
| anterior part of the Deep and periventricular white matter (left) | | | 0.020121 | 0.002172 | | | 0.018856 | | 0.002482 |
| anterior part of the Deep and periventricular white matter (right) | | | 0.019143 | 0.001931 | | | 0.018035 | | 0.002093 |
| posterior part of the Deep and periventricular white matter (left) | | | 0.001765 | 0.000186 | | | 0.001846 | | 0.000268 |
| posterior part of the Deep and periventricular white matter (right) | | | 0.002056 | 0.000235 | | | 0.002171 | | 0.000298 |
| Body of corpus callosum (left) | | | 0.00334 | 0.000394 | | | 0.003485 | | 0.000489 |
| Body of corpus callosum (right) | | | 0.003448 | 0.000416 | | | 0.003653 | | 0.000464 |
| Splenium of corpus callosum (left) | | | 0.004688 | 0.000546 | | | 0.004588 | | 0.000581 |
| Splenium of corpus callosum (right) | | | 0.004756 | 0.000553 | | | 0.004696 | | 0.0005 |
| posterior part of the periventricular area (left) | | | 0.001022 | 0.000417 | | | 0.001179 | | 0.000382 |
| posterior part of the periventricular area (right) | | | 0.000975 | 0.000415 | | | 0.001202 | | 0.00039 |
| Anterior limb of internal capsule (left) | | | 0.001895 | 0.000204 | | | 0.001934 | | 0.000168 |
| Anterior limb of internal capsule (right) | | | 0.001894 | 0.000175 | | | 0.001974 | | 0.000188 |
| Posterior limb of internal capsule (left) | | | 0.002266 | 0.00019 | | | 0.002239 | | 0.000185 |
| Posterior limb of internal capsule (right) | | | 0.002155 | 0.000182 | | | 0.002141 | | 0.000157 |
| inferior part of the Deep and periventricular white matter (left) | | | 0.014904 | 0.000826 | | | 0.01454 | | 0.00085 |
| inferior part of the Deep and periventricular white matter (right) | | | 0.013891 | 0.000874 | | | 0.01364 | | 0.000785 |
| Cingulum (cingulate gyrus part) (left) | | | 0.001947 | 0.000234 | | | 0.001918 | | 0.000221 |
| Cingulum (cingulate gyrus part) (right) | | | 0.002084 | 0.000241 | | | 0.00205 | | 0.000241 |
| Cingulum (hippocampal part) (left) | | | 0.001154 | 0.000147 | | | 0.001015 | | 0.000155 |
| Cingulum (hippocampal part) (right) | | | 0.00116 | 0.000113 | | | 0.001037 | | 0.000186 |
| Fornix/stria terminalis (left) | | | 0.000975 | 0.0001 | | | 0.000936 | | 8.81E-05 |
| Fornix/stria terminalis (right) | | | 0.00101 | 9.56E-05 | | | 0.000986 | | 8.9E-05 |
| Fornix (left) | | | 0.000497 | 6.75E-05 | | | 0.000559 | | 0.000106 |
| Fornix (right) | | | 0.000656 | 8.37E-05 | | | 0.000707 | | 0.000132 |
| peripheral parietal white matter (left) | | | 0.02613 | 0.002099 | | | 0.025816 | | 0.001859 |
| peripheral parietal white matter (right) | | | 0.027236 | 0.002107 | | | 0.026929 | | 0.001939 |
| Anterior part of the lateral ventricle (left) | | | 0.010539 | 0.004628 | | | 0.01368 | | 0.00562 |
| posterior part of the lateral ventricle (left) | | | 0.003297 | 0.001658 | | | 0.004802 | | 0.002853 |
| Inferior part of the lateral ventricle (left) | | | 0.001335 | 0.000529 | | | 0.001862 | | 0.000917 |
| Anterior part of the lateral ventricle (right) | | | 0.011056 | 0.004925 | | | 0.014519 | | 0.00526 |
| posterior part of the lateral ventricle (right) | | | 0.003263 | 0.001642 | | | 0.005089 | | 0.002641 |
| Inferior part of the lateral ventricle (right) | | | 0.00143 | 0.000721 | | | 0.002087 | | 0.000966 |
| Third and fourth ventricles | | | 0.004194 | 0.000834 | | | 0.004378 | | 0.000915 |
| anterior part of the periventricular area (left) | | | 0.001409 | 0.000508 | | | 0.001693 | | 0.000709 |
| anterior part of the periventricular area (right) | | | 0.001264 | 0.000488 | | | 0.001587 | | 0.000744 |
| peripheral frontal white matter (left) | | | 0.037694 | 0.002558 | | | 0.037862 | | 0.003108 |
| peripheral frontal white matter (right) | | | 0.038762 | 0.002424 | | | 0.038965 | | 0.003095 |
| peripheral temporal white matter (left) | | | 0.020923 | 0.001195 | | | 0.019958 | | 0.001716 |
| peripheral temporal white matter (right) | | | 0.021145 | 0.001279 | | | 0.019792 | | 0.00192 |
| peripheral occipital white matter (left) | | | 0.020998 | 0.001521 | | | 0.021174 | | 0.001773 |
| peripheral occipital white matter (right) | | | 0.020524 | 0.001579 | | | 0.020475 | | 0.001796 |
| subcortical white matter of the cingulate gyrus (left) | | | 0.003754 | 0.000363 | | | 0.003708 | | 0.000327 |
| subcortical white matter of the cingulate gyrus (right) | | | 0.003158 | 0.000317 | | | 0.003117 | | 0.000262 |
| cerebellum white matter (right) | | | 0.00909 | 0.001405 | | | 0.009446 | | 0.001 |
| cerebellum white matter (left) | | | 0.009263 | 0.00127 | | | 0.00958 | | 0.000819 |
| marrow | | | 0 | 0 | | | 0 | | 0 |
| sulci of the frontal lobe (left) | | | 0.02006 | 0.00267 | | | 0.021319 | | 0.002812 |
| sulci of the frontal lobe (right) | | | 0.016161 | 0.002298 | | | 0.01709 | | 0.002159 |
| Central sulcus (left) | | | 0.00276 | 0.000711 | | | 0.002804 | | 0.00075 |
| Central sulcus (right) | | | 0.002762 | 0.000722 | | | 0.00288 | | 0.00067 |
| Sylvian fissure and anterior insular sulcus (left) | | | 0.00269 | 0.000987 | | | 0.003281 | | 0.0013 |
| Sylvian fissure and anterior insular sulcus (right) | | | 0.003097 | 0.000906 | | | 0.003594 | | 0.001105 |
| Sylvian fissure and posterior insular sulcus (left) | | | 0.003784 | 0.000991 | | | 0.004386 | | 0.001172 |
| Sylvian fissure and posterior insular sulcus (right) | | | 0.003577 | 0.00094 | | | 0.004057 | | 0.00084 |
| Extension of Sylvian fissure into supramarginal gyrus (left) | | | 0.000313 | 0.000172 | | | 0.000406 | | 0.000251 |
| Extension of Sylvian fissure into supramarginal gyrus (right) | | | 0.000436 | 0.000223 | | | 0.000562 | | 0.000252 |
| sulci of the parietal lobe (left) | | | 0.008602 | 0.002017 | | | 0.008627 | | 0.001787 |
| sulci of the parietal lobe (right) | | | 0.00931 | 0.002187 | | | 0.0095 | | 0.001841 |
| sulci of the cingulate gyrus (left) | | | 0.004162 | 0.001142 | | | 0.00432 | | 0.001138 |
| sulci of the cingulate gyrus (right) | | | 0.003774 | 0.001045 | | | 0.004106 | | 0.001109 |
| sulci of the occipital lobe (left) | | | 0.003168 | 0.00119 | | | 0.003343 | | 0.001048 |
| sulci of the occipital lobe (right) | | | 0.003403 | 0.001168 | | | 0.003773 | | 0.001134 |
| Sulci of the temporal lobe (left) | | | 0.002344 | 0.000765 | | | 0.002746 | | 0.000611 |
| Sulci of the temporal lobe (right) | | | 0.001862 | 0.000644 | | | 0.002218 | | 0.000565 |
| Level5 | | | | | | | | | |
| Superior frontal gyrus (left) | | 0.008101 | | 0.000948 | | | 0.007841 | | 0.000914 |
| Superior frontal gyrus (right) | | 0.007109 | | | 0.000859 | 0.006812 | | 0.000812 | |
| superior frontal gyrus/ prefrontal cortex (left) | | 0.007725 | | 0.000691 | | | 0.007554 | | 0.000883 |
| superior frontal gyrus/ prefrontal cortex (right) | | 0.006721 | | 0.000682 | | | 0.006519 | | 0.000819 |
| superior frontal gyrus/ pole (left) | | 0.001939 | | 0.000219 | | | 0.001973 | | 0.000292 |
| superior frontal gyru/ pole (right) | | 0.002604 | | 0.000308 | | | 0.002548 | | 0.000304 |
| Middle frontal gyrus (left) | | 0.006749 | | 0.000799 | | | 0.006474 | | 0.000733 |
| Middle frontal gyrus (right) | | 0.006942 | | 0.000851 | | | 0.006541 | | 0.000705 |
| Middle frontal gyrus (dorsolateral prefrontal cortex) (left) | | 0.009287 | | 0.000925 | | | 0.008946 | | 0.000995 |
| Middle frontal gyrus (dorsolateral prefrontal cortex) (right) | | 0.009327 | | 0.001121 | | | 0.00913 | | 0.000946 |
| inferior frontal gyrus/pars opercularis (left) | | 0.002541 | | 0.000315 | | | 0.002482 | | 0.000348 |
| inferior frontal gyrus/pars opercularis (right) | | 0.002362 | | 0.000368 | | | 0.002343 | | 0.000392 |
| inferior frontal gyrus/pars orbitalis (left) | | 0.002648 | | 0.000341 | | | 0.002576 | | 0.000283 |
| inferior frontal gyrus/pars orbitalis (right) | | 0.00236 | | 0.000289 | | | 0.002364 | | 0.00036 |
| inferior frontal gyrus/ pars triangularis (left) | | 0.00254 | | 0.000412 | | | 0.002463 | | 0.000393 |
| inferior frontal gyrus/ pars triangularis (right) | | 0.002279 | | 0.000345 | | | 0.002179 | | 0.0003 |
| LATERAL FRONTO-ORBITAL GYRUS (left) | | 0.003453 | | 0.000319 | | | 0.003361 | | 0.000322 |
| LATERAL FRONTO-ORBITAL GYRUS (right) | | 0.003419 | | 0.000342 | | | 0.003277 | | 0.000314 |
| MIDDLE FRONTO-ORBITAL GYRUS (left) | | 0.00343 | | 0.000358 | | | 0.003417 | | 0.00036 |
| MIDDLE FRONTO-ORBITAL GYRUS (right) | | 0.003043 | | 0.000316 | | | 0.00296 | | 0.000336 |
| GYRUS RECTUS (left) | | 0.003717 | | 0.000421 | | | 0.003603 | | 0.00048 |
| GYRUS RECTUS (right) | | 0.003667 | | 0.000384 | | | 0.003569 | | 0.000512 |
| Postcentral gyrus (left) | | 0.008347 | | 0.000931 | | | 0.008184 | | 0.000809 |
| Postcentral gyrus (right) | | 0.008412 | | 0.000768 | | | 0.008479 | | 0.000782 |
| Precentral gyrus (left) | | 0.009724 | | 0.001014 | | | 0.010055 | | 0.001075 |
| Precentral gyrus (right) | | 0.011088 | | 0.0011 | | | 0.011276 | | 0.000946 |
| SUPERIOR PARIETAL LOBULE (left) | | 0.006386 | | 0.000942 | | | 0.006337 | | 0.000956 |
| SUPERIOR PARIETAL LOBULE (right) | | 0.005509 | | 0.000964 | | | 0.005315 | | 0.001135 |
| Supramarginal Gyrus (left) | | 0.006537 | | 0.00098 | | | 0.006244 | | 0.000888 |
| Supramarginal Gyrus (right) | | 0.006829 | | 0.000732 | | | 0.00672 | | 0.000788 |
| angular gyrus (left) | | 0.005543 | | 0.000638 | | | 0.005239 | | 0.000664 |
| angular gyrus (right) | | 0.008901 | | 0.000906 | | | 0.00862 | | 0.001004 |
| Precuneus (left) | | 0.005001 | | 0.000522 | | | 0.004796 | | 0.000492 |
| Precuneus (right) | | 0.004614 | | 0.000539 | | | 0.004343 | | 0.000658 |
| superior temporal gyrus (left) | | 0.008818 | | 0.000852 | | | 0.008246 | | 0.000847 |
| superior temporal gyrus (right) | | 0.008355 | | 0.000986 | | | 0.007725 | | 0.00085 |
| superior temporal gyrus/ pole (left) | | 0.003585 | | 0.000452 | | | 0.003233 | | 0.000448 |
| superior temporal gyrus/ pole (right) | | 0.003702 | | 0.000468 | | | 0.003274 | | 0.000621 |
| Middle temporal gyrus (left) | | 0.012013 | | 0.001056 | | | 0.011229 | | 0.001182 |
| Middle temporal gyrus (right) | | 0.009677 | | 0.001161 | | | 0.008776 | | 0.001122 |
| Middle temporal gyrus＿pole (left) | | 0.001883 | | 0.000272 | | | 0.001771 | | 0.000325 |
| Middle temporal gyrus＿pole (right) | | 0.001757 | | 0.000326 | | | 0.001572 | | 0.000427 |
| inferior temporal gyrus (left) | | 0.008885 | | 0.000863 | | | 0.00803 | | 0.000874 |
| inferior temporal gyrus (right) | | 0.009735 | | 0.000982 | | | 0.008561 | | 0.001257 |
| Parahippocampal gyrus (left) | | 0.000895 | | 9.77E-05 | | | 0.000794 | | 1.07E-04 |
| Parahippocampal gyrus (right) | | 0.000939 | | 0.000113 | | | 0.000846 | | 0.000128 |
| ENTORHINAL AREA (left) | | 0.000672 | | 7.94E-05 | | | 0.000611 | | 1.19E-04 |
| ENTORHINAL AREA (right) | | 0.000882 | | 0.000125 | | | 0.000802 | | 0.000186 |
| Fusiform gyrus (left) | | 0.01115 | | 0.000724 | | | 0.010662 | | 0.000818 |
| Fusiform gyrus (right) | | 0.010974 | | 0.000799 | | | 0.010381 | | 0.001054 |
| SUPERIOR OCCIPITAL GYRUS (left) | | 0.002194 | | 0.000462 | | | 0.002124 | | 0.000472 |
| SUPERIOR OCCIPITAL GYRUS (right) | | 0.001917 | | 0.000426 | | | 0.001857 | | 0.000334 |
| middle occipital gyrus (left) | | 0.011366 | | 0.001316 | | | 0.011156 | | 0.001319 |
| middle occiptial gyrus (right) | | 0.011805 | | 0.00132 | | | 0.011443 | | 0.001555 |
| inferior occipital gyrus (left) | | 0.003324 | | 0.000485 | | | 0.003269 | | 0.000465 |
| inferior occipital gyrus (right) | | 0.004376 | | 0.000692 | | | 0.004226 | | 0.000615 |
| Cuneus (left) | | 0.004886 | | 0.000484 | | | 0.004973 | | 0.000749 |
| Cuneus (right) | | 0.00383 | | 0.000484 | | | 0.003888 | | 0.000616 |
| Lingual gyrus (left) | | 0.006429 | | 0.000634 | | | 0.006488 | | 0.000728 |
| Lingual gyrus (right) | | 0.005598 | | 0.000594 | | | 0.005792 | | 0.000652 |
| rostral_Anterior cingulate cortex (left) | | 0.00152 | | 0.000228 | | | 0.001498 | | 0.000222 |
| rostral_Anterior cingulate cortex (right) | | 0.001606 | | 0.000262 | | | 0.001673 | | 0.000278 |
| subcallosal_Anterior cingulate cortex (left) | | 0.000254 | | 6.46E-05 | | | 0.000278 | | 5.71E-05 |
| subcallosal_Anterior cingulate cortex (right) | | 0.000366 | | 6.76E-05 | | | 0.000375 | | 6.10E-05 |
| subgenual anterior cingulate cortex (left) | | 0.001307 | | 0.000132 | | | 0.001314 | | 0.000146 |
| subgenual anterior cingulate cortex (right) | | 0.001236 | | 0.000145 | | | 0.001274 | | 0.000145 |
| dorsal Anterior cingulate cortex (left) | | 0.007628 | | 0.00061 | | | 0.007544 | | 0.00061 |
| dorsal Anterior cingulate cortex (right) | | 0.007184 | | 0.000624 | | | 0.007071 | | 0.000557 |
| Posterior cingulate cortex (left) | | 0.00503 | | 0.000568 | | | 0.004885 | | 0.000619 |
| Posterior cingulate cortex (right) | | 0.005791 | | 0.000618 | | | 0.005594 | | 0.000629 |
| Insula (left) | | 0.004783 | | 0.000484 | | | 0.004468 | | 0.000479 |
| Insula (right) | | 0.005142 | | 0.000508 | | | 0.004809 | | 0.000437 |
| Amygdala (left) | | 0.001214 | | 0.000143 | | | 0.001002 | | 0.000146 |
| Amygdala (right) | | 0.001334 | | 0.000145 | | | 0.001097 | | 0.000159 |
| hippocampus (left) | | 0.002573 | | 0.000241 | | | 0.002319 | | 0.000283 |
| hippocampus (right) | | 0.002691 | | 0.000261 | | | 0.002373 | | 0.000303 |
| Caudate nucleus (left) | | 0.002773 | | 0.00027 | | | 0.002768 | | 0.000338 |
| Caudate nucleus (right) | | 0.002804 | | 0.000311 | | | 0.002806 | | 0.000366 |
| putamen (left) | | 0.00296 | | 0.000295 | | | 0.002796 | | 0.000381 |
| putamen (right) | | 0.003146 | | 0.000338 | | | 0.002975 | | 0.000391 |
| Globus Pallidus (left) | | 0.001064 | | 0.000125 | | | 0.001061 | | 0.000117 |
| Globus Pallidus (right) | | 0.001141 | | 0.000128 | | | 0.001141 | | 0.000137 |
| thalamus (left) | | 0.003953 | | 0.000331 | | | 0.003877 | | 0.000316 |
| thalamus (right) | | 0.004007 | | 0.00036 | | | 0.003895 | | 0.000323 |
| Hypothalamus (left) | | 0.000447 | | 4.05E-05 | | | 0.00044 | | 4.10E-05 |
| Hypothalamus (right) | | 0.000519 | | 4.75E-05 | | | 0.000507 | | 4.68E-05 |
| Nucleus basalis of Meynert (left) | | 0.001914 | | 0.000165 | | | 0.001841 | | 0.000183 |
| Nucleus basalis of Meynert (right) | | 0.000797 | | 0.000102 | | | 0.000779 | | 0.000109 |
| Nucleus accumbens (left) | | 0.000443 | | 5.48E-05 | | | 0.000407 | | 7.67E-05 |
| Nucleus accumbens (right) | | 0.000568 | | 6.76E-05 | | | 0.000536 | | 1.06E-04 |
| red nucleus (left) | | 0.000232 | | 3.15E-05 | | | 0.00023 | | 3.72E-05 |
| red nucleus (right) | | 0.000262 | | 4.47E-05 | | | 0.00027 | | 3.82E-05 |
| substantia nigra (left) | | 0.000233 | | 4.30E-05 | | | 0.000231 | | 3.36E-05 |
| substantia nigra (right) | | 0.00019 | | 2.97E-05 | | | 0.000184 | | 3.00E-05 |
| cerebellum gray matter (right) | | 0.037611 | | 0.004981 | | | 0.038365 | | 0.00404 |
| cerebellum gray matter (left) | | 0.038307 | | 0.004518 | | | 0.039282 | | 0.003273 |
| Cerebral peduncle (left) | | 0.001584 | | 0.000147 | | | 0.001502 | | 0.000127 |
| Cerebral peduncle (right) | | 0.001581 | | 0.000134 | | | 0.00149 | | 0.000126 |
| midbrain (left) | | 0.001872 | | 0.000158 | | | 0.001839 | | 0.00018 |
| midbrain (right) | | 0.00178 | | 0.00015 | | | 0.001742 | | 0.000166 |
| corticospinal tract (left) | | 0.001247 | | 0.00017 | | | 0.001256 | | 0.000177 |
| corticospinal tract (right) | | 0.001181 | | 0.000162 | | | 0.001161 | | 0.000156 |
| superior cerebellar peduncle (left) | | 0.000668 | | 6.40E-05 | | | 0.00065 | | 7.11E-05 |
| superior cerebellar peduncle (right) | | 0.000737 | | 7.04E-05 | | | 0.000723 | | 8.66E-05 |
| middle cerebellar peduncle (left) | | 0.002584 | | 0.0003 | | | 0.002455 | | 0.000294 |
| middle cerebellar peduncle (right) | | 0.002815 | | 0.000316 | | | 0.002688 | | 0.000305 |
| Pontine crossing tract (left) | | 0.000669 | | 0.000102 | | | 0.000664 | | 0.000109 |
| Pontine crossing tract (right) | | 0.000702 | | 0.000117 | | | 0.000681 | | 0.000106 |
| Inferior cerebellar peduncle (left) | | 0.000554 | | 0.000113 | | | 0.000562 | | 6.66E-05 |
| Inferior cerebellar peduncle (right) | | 0.000568 | | 0.000116 | | | 0.000581 | | 9.22E-05 |
| medial lemniscus (left) | | 0.000569 | | 0.000108 | | | 0.000571 | | 8.69E-05 |
| medial lemniscus (right) | | 0.000714 | | 0.000104 | | | 0.0007 | | 0.000106 |
| pons (left) | | 0.000213 | | 3.72E-05 | | | 0.000207 | | 4.18E-05 |
| pons (right) | | 0.000477 | | 8.23E-05 | | | 0.00045 | | 8.98E-05 |
| medulla (left) | | 0.001148 | | 0.000242 | | | 0.001193 | | 0.000178 |
| medulla (right) | | 0.001287 | | 0.000265 | | | 0.001291 | | 0.000202 |
| Anterior corona radiata (left) | | 0.008691 | | 0.001123 | | | 0.008051 | | 0.001493 |
| Anterior corona radiata (right) | | 0.008194 | | 0.000969 | | | 0.007665 | | 0.001217 |
| Superior corona radiata (left) | | 0.01118 | | 0.001313 | | | 0.010537 | | 0.001138 |
| Superior corona radiata (right) | | 0.010747 | | 0.001238 | | | 0.010161 | | 0.001051 |
| Posterior corona radiata (left) | | 0.004037 | | 0.000524 | | | 0.003844 | | 0.000435 |
| Posterior corona radiata (right) | | 0.003419 | | 0.000468 | | | 0.003168 | | 0.000419 |
| Genu of corpus callosum (left) | | 0.001765 | | 0.000186 | | | 0.001846 | | 0.000268 |
| Genu of corpus callosum (right) | | 0.002056 | | 0.000235 | | | 0.002171 | | 0.000298 |
| Body of corpus callosum (left) | | 0.00334 | | 0.000394 | | | 0.003485 | | 0.000489 |
| Body of corpus callosum (right) | | 0.003448 | | 0.000416 | | | 0.003653 | | 0.000464 |
| Splenium of corpus callosum (left) | | 0.004688 | | 0.000546 | | | 0.004588 | | 0.000581 |
| Splenium of corpus callosum (right) | | 0.004756 | | 0.000553 | | | 0.004696 | | 0.0005 |
| lateral part of the periventricular white matter (left) | | 0.000483 | | 0.000322 | | | 0.000555 | | 0.000243 |
| lateral part of the periventricular white matter (right) | | 0.000407 | | 0.000315 | | | 0.0005 | | 0.000257 |
| Anterior limb of internal capsule (left) | | 0.001895 | | 0.000204 | | | 0.001934 | | 0.000168 |
| Anterior limb of internal capsule (right) | | 0.001894 | | 0.000175 | | | 0.001974 | | 0.000188 |
| Posterior limb of internal capsule (left) | | 0.002266 | | 0.00019 | | | 0.002239 | | 0.000185 |
| Posterior limb of internal capsule (right) | | 0.002155 | | 0.000182 | | | 0.002141 | | 0.000157 |
| retrolenticular part of internal capsule (left) | | 0.002047 | | 0.000181 | | | 0.00201 | | 0.000174 |
| retrolenticular part of internal capsule (right) | | 0.002359 | | 0.000231 | | | 0.002347 | | 0.000158 |
| external capsule (left) | | 0.002438 | | 0.000201 | | | 0.002404 | | 0.000284 |
| external capsule (right) | | 0.002465 | | 0.00022 | | | 0.002444 | | 0.00026 |
| Cingulum (cingulate gyrus part) (left) | | 0.001947 | | 0.000234 | | | 0.001918 | | 0.000221 |
| Cingulum (cingulate gyrus part) (right) | | 0.002084 | | 0.000241 | | | 0.00205 | | 0.000241 |
| Cingulum (hippocampal part) (left) | | 0.001154 | | 0.000147 | | | 0.001015 | | 0.000155 |
| Cingulum (hippocampal part) (right) | | 0.00116 | | 0.000113 | | | 0.001037 | | 0.000186 |
| Fornix/stria terminalis (left) | | 0.000975 | | 0.0001 | | | 0.000936 | | 8.81E-05 |
| Fornix/stria terminalis (right) | | 0.00101 | | 9.56E-05 | | | 0.000986 | | 8.90E-05 |
| Fornix (left) | | 0.000497 | | 6.75E-05 | | | 0.000559 | | 1.06E-04 |
| Fornix (right) | | 0.000656 | | 8.37E-05 | | | 0.000707 | | 1.32E-04 |
| Inferior fronto-occipital fasciculus (left) | | 0.001516 | | 0.000148 | | | 0.001424 | | 0.000291 |
| Inferior fronto-occipital fasciculus (right) | | 0.001497 | | 0.000177 | | | 0.001443 | | 0.000205 |
| Posterior thalamic radiation (left) | | 0.006443 | | 0.000471 | | | 0.006275 | | 0.000561 |
| Posterior thalamic radiation (right) | | 0.004632 | | 0.000395 | | | 0.004571 | | 0.000433 |
| Sagittal stratum (left) | | 0.002461 | | 0.000256 | | | 0.002405 | | 0.000174 |
| Sagittal stratum (right) | | 0.002938 | | 0.000283 | | | 0.002836 | | 0.000297 |
| Superior fronto-occipital fascicul (left) | | 0.000248 | | 6.09E-05 | | | 0.00027 | | 5.04E-05 |
| Superior fronto-occipital fascicul (right) | | 0.000203 | | 6.42E-05 | | | 0.00021 | | 4.50E-05 |
| Superior longitudinal fasciculus (left) | | 0.004716 | | 0.000778 | | | 0.00429 | | 0.000705 |
| Superior longitudinal fasciculus (right) | | 0.005585 | | 0.000955 | | | 0.005096 | | 0.000745 |
| claustrum (left) | | 0.000285 | | 4.89E-05 | | | 0.000249 | | 4.10E-05 |
| claustrum (right) | | 0.0003 | | 3.68E-05 | | | 0.00025 | | 3.62E-05 |
| Mammillary body (right) | | 6.80E-05 | | 1.12E-05 | | | 6.72E-05 | | 1.28E-05 |
| Mammillary body (left) | | 7.42E-05 | | 9.79E-06 | | | 7.08E-05 | | 1.52E-05 |
| optic tract (left) | | 0 | | 0 | | | 0 | | 0 |
| optic tract (right) | | 0 | | 0 | | | 0 | | 0 |
| frontal horn of the lateral ventricle (left) | | 0.005463 | | 0.002377 | | | 0.007051 | | 0.002787 |
| body of the lateral ventricle (left) | | 0.004754 | | 0.002412 | | | 0.006268 | | 0.002966 |
| Lateral ventricle, atrium part (left) | | 0.002747 | | 0.001434 | | | 0.004064 | | 0.002546 |
| Occipital horn of the lateral ventricle (left) | | 0.000551 | | 0.000298 | | | 0.000738 | | 0.000395 |
| inferior horn of the lateral ventricle (left) | | 0.001335 | | 0.000529 | | | 0.001862 | | 0.000917 |
| frontal horn of the lateral ventricle (right) | | 0.006084 | | 0.002592 | | | 0.007751 | | 0.002731 |
| body of the lateral ventricle (right) | | 0.00465 | | 0.002446 | | | 0.006375 | | 0.002622 |
| Lateral ventricle, atrium part (right) | | 0.002849 | | 0.001489 | | | 0.00453 | | 0.002458 |
| Occipital horn of the lateral ventricle (right) | | 0.000414 | | 0.000208 | | | 0.000559 | | 0.000253 |
| inferior horn of the lateral ventricle (right) | | 0.00143 | | 0.000721 | | | 0.002087 | | 0.000966 |
| Third and fourth ventricles | | 0.004194 | | 0.000834 | | | 0.004378 | | 0.000915 |
| anterior part of the periventricular white matter (left) | | 0.001409 | | 0.000508 | | | 0.001693 | | 0.000709 |
| anterior part of the periventricular white matter (right) | | 0.001264 | | 0.000488 | | | 0.001587 | | 0.000744 |
| posterior part of the periventricular white matter (left) | | 0.000533 | | 0.000131 | | | 0.000624 | | 0.000165 |
| posterior part of the periventricular white matter (right) | | 0.000568 | | 0.000129 | | | 0.000692 | | 0.000177 |
| subcortical white matter of the superior frontal gyrus (left) | | 0.005832 | | 0.000675 | | | 0.005771 | | 0.000753 |
| subcortical white matter of the superior frontal gyrus (right) | | 0.006107 | | 0.000733 | | | 0.006213 | | 0.000645 |
| subcortical white matter of the superior frontal gyrus/ prefrontal cortex (left) | | 0.005089 | | 0.000626 | | | 0.004986 | | 0.000722 |
| subcortical white matter of the superior frontal gyrus/ prefrontal cortex (right) | | 0.004725 | | 0.000603 | | | 0.004735 | | 0.000809 |
| subcortical white matter of the superior frontal gyrus/ pole (left) | | 0.000243 | | 4.63E-05 | | | 0.00025 | | 5.39E-05 |
| subcortical white matter of the superior frontal gyrus/ pole (right) | | 0.000318 | | 7.57E-05 | | | 0.000336 | | 6.38E-05 |
| subcortical white matter of the middle frontal gyrus (left) | | 0.004257 | | 0.00056 | | | 0.004184 | | 0.000652 |
| subcortical white matter of the middle frontal gyrus (right) | | 0.004213 | | 0.000545 | | | 0.004008 | | 0.000527 |
| subcortical white matter of the middle frontal gyrus/ dorsolateral prefrontal cortex (left) | | 0.002069 | | 0.000304 | | | 0.001979 | | 0.000435 |
| subcortical white matter of the middle frontal gyrus/ dorsolateral prefrontal cortex (right) | | 0.002246 | | 0.000379 | | | 0.002189 | | 0.000444 |
| subcortical white matter of the inferior frontal gyrus/pars opecularis (left) | | 0.001444 | | 0.000226 | | | 0.001495 | | 0.000301 |
| subcortical white matter of the inferior frontal gyrus/pars opecularis (right) | | 0.001626 | | 0.000309 | | | 0.00159 | | 0.000338 |
| subcortical white matter of the inferior frontal gyrus/pars orbitalis (left) | | 0.001782 | | 0.000288 | | | 0.001776 | | 0.000252 |
| subcortical white matter of the inferior frontal gyrus/pars orbitalis (right) | | 0.0018 | | 0.00024 | | | 0.001735 | | 0.000304 |
| subcortical white matter of the inferior frontal gyrus/pars triangularis (left) | | 0.002103 | | 0.000347 | | | 0.001999 | | 0.000349 |
| subcortical white matter of the inferior frontal gyrus/pars triangularis (right) | | 0.00158 | | 0.000262 | | | 0.001491 | | 0.000261 |
| subcortical white matter of the lateral fronto-orbital gyrus (left) | | 0.001384 | | 0.000175 | | | 0.001462 | | 0.000182 |
| subcortical white matter of the lateral fronto-orbital gyrus (right) | | 0.001402 | | 0.000201 | | | 0.001411 | | 0.000201 |
| subcortical white matter of the middle fronto-orbital gyrus (left) | | 0.001641 | | 0.00024 | | | 0.001649 | | 0.000218 |
| subcortical white matter of the middle fronto-orbital gyrus (right) | | 0.00131 | | 0.000224 | | | 0.001352 | | 0.000255 |
| subcortical white matter of the gyrus rectus (left) | | 0.001438 | | 0.000242 | | | 0.001528 | | 0.000252 |
| subcortical white matter of the gyrus rectus (right) | | 0.001756 | | 0.000283 | | | 0.001835 | | 0.000332 |
| subcortical white matter of the postcentral gyrus (left) | | 0.007956 | | 0.000858 | | | 0.007986 | | 0.000935 |
| subcortical white matter of the postcentral gyrus (right) | | 0.008048 | | 0.000877 | | | 0.008171 | | 0.000763 |
| subcortical white matter of the precentral gyrus (left) | | 0.010417 | | 0.001019 | | | 0.010786 | | 0.001046 |
| subcortical white matter of the precentral gyrus (right) | | 0.011678 | | 0.001109 | | | 0.012073 | | 0.001107 |
| subcortical white matter of the superior parietal lobule (left) | | 0.006274 | | 0.000783 | | | 0.006258 | | 0.000796 |
| subcortical white matter of the superior parietal lobule (right) | | 0.005939 | | 0.000746 | | | 0.005921 | | 0.000824 |
| subcortical white matter of the supramarginal gyrus (left) | | 0.002848 | | 0.000537 | | | 0.002864 | | 0.000434 |
| subcortical white matter of the supramarginal gyrus (right) | | 0.003004 | | 0.000393 | | | 0.003008 | | 0.000421 |
| Angular white matter (left) | | 0.002312 | | 0.000443 | | | 0.002311 | | 0.000355 |
| Anterior white matter (right) | | 0.002678 | | 0.000439 | | | 0.002668 | | 0.00037 |
| subcortical white matter of the precuneus (left) | | 0.002025 | | 0.000298 | | | 0.002109 | | 0.000335 |
| subcortical white matter of the precuneus (right) | | 0.001983 | | 0.000263 | | | 0.002067 | | 0.000332 |
| subcortical white matter of the superior temporal gyrus (left) | | 0.006405 | | 0.000605 | | | 0.006065 | | 0.000555 |
| subcortical white matter of the superior temporal gyrus (right) | | 0.006634 | | 0.000774 | | | 0.00613 | | 0.00059 |
| subcortical white matter of the superior temporal gyrus/ pole (left) | | 0.001224 | | 0.000231 | | | 0.001264 | | 0.00026 |
| subcortical white matter of the superior temporal gyrus/ pole (right) | | 0.001264 | | 0.000213 | | | 0.001264 | | 0.000284 |
| subcortical white matter of the middle temporal gyrus (left) | | 0.005673 | | 0.000481 | | | 0.005244 | | 0.000639 |
| subcortical white matter of the middle temporal gyrus (right) | | 0.005284 | | 0.000609 | | | 0.004757 | | 0.000635 |
| subcortical white matter of the middle temporal gyrus/ pole (left) | | 0.000932 | | 0.000169 | | | 0.000946 | | 0.000281 |
| subcortical white matter of the middle temporal gyrus/ pole (right) | | 0.000869 | | 0.000204 | | | 0.000852 | | 0.000288 |
| subcortical white matter of the inferior temporal gyrus (left) | | 0.003427 | | 0.000428 | | | 0.003277 | | 0.000558 |
| subcortical white matter of the inferior temporal gyrus (right) | | 0.003411 | | 0.000373 | | | 0.003171 | | 0.000582 |
| subcortical white matter of the fusiform gyrus (left) | | 0.003264 | | 0.000418 | | | 0.003165 | | 0.000306 |
| subcortical white matter of the fusiform gyrus (right) | | 0.003675 | | 0.00036 | | | 0.003619 | | 0.000499 |
| subcortical white matter of the superior occipital gyrus (left) | | 0.002596 | | 0.000396 | | | 0.002498 | | 0.000351 |
| subcortical white matter of the superior occipital gyrus (right) | | 0.003075 | | 0.000497 | | | 0.003012 | | 0.000381 |
| subcortical white matter of the middle occiptial gyrus (left) | | 0.007537 | | 0.000927 | | | 0.007505 | | 0.000962 |
| subcortical white matter of the middle occiptial gyrus (right) | | 0.008121 | | 0.001034 | | | 0.007892 | | 0.001249 |
| subcortical wihte matter of the inferior occipital gyrus (left) | | 0.001377 | | 0.000274 | | | 0.00137 | | 0.000262 |
| subcortical white matter of the inferior occipital gyrus (right) | | 0.001981 | | 0.000341 | | | 0.00185 | | 0.000318 |
| subcortical white matter of the cuneus (left) | | 0.004064 | | 0.000544 | | | 0.004063 | | 0.000568 |
| subcortical white matter of the cuneus (right) | | 0.002714 | | 0.00039 | | | 0.002822 | | 0.000408 |
| subcortical white matter of the lingual gyrus (left) | | 0.005424 | | 0.000495 | | | 0.00574 | | 0.000586 |
| subcortical white matter of the lingual gyrus (right) | | 0.004634 | | 0.000516 | | | 0.004901 | | 0.000582 |
| subcortical white matter of the rostral anterior cingulate cortex (left) | | 8.67E-05 | | 5.35E-05 | | | 8.18E-05 | | 5.15E-05 |
| subcortical white matter of the rostral anterior cingulate cortex (right) | | 4.11E-07 | | 1.22E-06 | | | 6.12E-07 | | 2.11E-06 |
| subcortical white matter of the subcallosal anterior cingulate cortex (left) | | 6.25E-05 | | 2.35E-05 | | | 6.39E-05 | | 2.47E-05 |
| subcortical white matter of the subcallosal anterior cingulate cortex (right) | | 6.20E-05 | | 3.01E-05 | | | 5.84E-05 | | 3.03E-05 |
| subcortical white matter of the subgenual anterior cingulate cortex (left) | | 0.000187 | | 3.29E-05 | | | 0.000187 | | 4.28E-05 |
| subcortical white matter of the subgenual anterior cingulate cortex (right) | | 9.85E-05 | | 2.42E-05 | | | 0.000107 | | 2.12E-05 |
| subcortical white matter of the dorsal anterior cingulate cortex (left) | | 0.001223 | | 0.000225 | | | 0.001265 | | 0.00017 |
| subcortical white matter of the dorsal anterior cingulate cortex (right) | | 0.000839 | | 0.000121 | | | 0.000907 | | 0.000109 |
| subcortical white matter of the postrior cingulate cortex (left) | | 0.00217 | | 0.000244 | | | 0.002107 | | 0.000242 |
| subcortical white matter of the posterior cingulate cortex (right) | | 0.002157 | | 0.000272 | | | 0.002041 | | 0.000232 |
| cerebellum white matter (right) | | 0.007361 | | 0.001166 | | | 0.007744 | | 0.00093 |
| cerebellum white matter (left) | | 0.007262 | | 0.001078 | | | 0.007611 | | 0.000782 |
| cerebrospinal fluid | | 0 | | 0 | | | 0 | | 0 |
| skull | | 0 | | 0 | | | 0 | | 0 |
| skull | | 0 | | 0 | | | 0 | | 0 |
| skull | | 0 | | 0 | | | 0 | | 0 |
| middle cerebellar peduncle in the cerebellum (left) | | 0.002001 | | 0.000385 | | | 0.001969 | | 0.000278 |
| middle cerebellar peduncle in the cerebellum (right) | | 0.00173 | | 0.000356 | | | 0.001702 | | 0.000252 |
| Bone marrow | | 0 | | 0 | | | 0 | | 0 |
| Inferior cerebellar peduncle/pons (left) | | 0.000393 | | 5.97E-05 | | | 0.00038 | | 5.13E-05 |
| Inferior cerebellar peduncle/pons (right) | | 0.000437 | | 5.74E-05 | | | 0.000428 | | 6.00E-05 |
| sulci of the frontal lobe (left) | | 0.02006 | | 0.00267 | | | 0.021319 | | 0.002812 |
| sulci of the frontal lobe (right) | | 0.016161 | | 0.002298 | | | 0.01709 | | 0.002159 |
| Central sulcus (left) | | 0.00276 | | 0.000711 | | | 0.002804 | | 0.00075 |
| Central sulcus (right) | | 0.002762 | | 0.000722 | | | 0.00288 | | 0.00067 |
| Sylvian fissure and anterior insular sulcus (left) | | 0.00269 | | 0.000987 | | | 0.003281 | | 0.0013 |
| Sylvian fissure and anterior insular sulcus (right) | | 0.003097 | | 0.000906 | | | 0.003594 | | 0.001105 |
| Sylvian fissure and posterior insular sulcus (left) | | 0.003784 | | 0.000991 | | | 0.004386 | | 0.001172 |
| Sylvian fissure and posterior insular sulcus (right) | | 0.003577 | | 0.00094 | | | 0.004057 | | 0.00084 |
| Extension of Sylvian fissure into supramarginal gyrus (left) | | 0.000313 | | 0.000172 | | | 0.000406 | | 0.000251 |
| Extension of Sylvian fissure into supramarginal gyrus (right) | | 0.000436 | | 0.000223 | | | 0.000562 | | 0.000252 |
| sulci of the parietal lobe (left) | | 0.008602 | | 0.002017 | | | 0.008627 | | 0.001787 |
| sulci of the parietal lobe (right) | | 0.00931 | | 0.002187 | | | 0.0095 | | 0.001841 |
| sulci of the cingulate gyrus (left) | | 0.004162 | | 0.001142 | | | 0.00432 | | 0.001138 |
| sulci of the cingulate gyrus (right) | | 0.003774 | | 0.001045 | | | 0.004106 | | 0.001109 |
| sulci of the occipital lobe (left) | | 0.003168 | | 0.00119 | | | 0.003343 | | 0.001048 |
| sulci of the occipital lobe (right) | | 0.003403 | | 0.001168 | | | 0.003773 | | 0.001134 |
| Sulci of the temporal lobe (left) | | 0.002344 | | 0.000765 | | | 0.002746 | | 0.000611 |
| Sulci of the temporal lobe (right) | | 0.001862 | | 0.000644 | | | 0.002218 | | 0.000565 |
| Caudate tail (left) | | 0 | | 0 | | | 0 | | 0 |
| Fimbria (left) | | 4.96E-06 | | 8.74E-06 | | | 4.32E-06 | | 7.73E-06 |
| Caudate tail (right) | | 0 | | 0 | | | 0 | | 0 |
| Fimbria (right) | | 5.52E-06 | | 8.09E-06 | | | 5.14E-06 | | 9.16E-06 |
| Choroid plexus of the lateral ventricle etc (left) | | 0.000322 | | 7.99E-05 | | | 0.000362 | | 9.10E-05 |
| Choroid plexus of the lateral ventricle etc (right) | | 0.000323 | | 8.28E-05 | | | 0.000394 | | 9.70E-05 |
